# HIF-1α suppresses SNPH expression to facilitate liver metastasis of colorectal cancer through regulating mitochondrial dynamics and filopodia formation

**DOI:** 10.1038/s41419-026-08551-1

**Published:** 2026-03-26

**Authors:** Lei Zhan, Xiaoxi Li, Xiaoyan Li, Qian Fei, Yue Jin, Jiaxing Yu, Luyao Tian, Feifei Li, Chunning Li, Qian Dong, Yong Zhang, Shulan Sun, Jingdong Zhang

**Affiliations:** 1 Medical Oncology Department of Gastrointestinal Cancer, Cancer Hospital of China Medical University, Liaoning Cancer Hospital & Institute, Cancer Hospital of Dalian University of Technology, Shenyang, Liaoning China; 2 Central Laboratory, Cancer Hospital of China Medical University, Liaoning Cancer Hospital & Institute, Cancer Hospital of Dalian University of Technology, Shenyang, China; 3 Department of Pathology, Cancer Hospital of China Medical University, Liaoning Cancer Hospital & Institute, Cancer Hospital of Dalian University of Technology, Shenyang, Liaoning China; 4 Department of Oncology, Shengjing Hospital of China Medical University, Shenyang, Liaoning China; 5 Liaoning Key Laboratory of Gastrointestinal Cancer Translational Research, Shenyang, Liaoning China

**Keywords:** Colorectal cancer, Metastasis, Tumour-suppressor proteins

## Abstract

Colorectal cancer (CRC) is a leading cause of cancer-associated deaths, with liver metastases developing in about 50% of patients. Mitochondrial dynamics play critical roles in a diverse range of cellular functions, including cell migration and cancer metastasis. However, the influence of mitochondrial dynamics deregulation in CRC liver metastasis is incompletely understood. Through multiple transcriptomic data analysis and validation, we found that low expression of *SNPH* significantly correlated with poor prognosis of CRC patients. SNPH knockdown altered mitochondrial dynamics to increase cell migration and invasion by promoting filopodia formation. Moreover, the reduced levels of SNPH were linked to HIF-1α expression. Luciferase reporter assay revealed that HIF-1α transcriptionally activated miR-130a-3p expression, which targeted SNPH mRNA to inhibit its protein levels. Furthermore, miR-130a-3p inhibitor suppressed SNPH downregulation, filopodia formation, and CRC cells metastasis under hypoxic conditions. Mechanistically, SNPH downregulation promoted ROS production, resulting in the activation of the AKT/cdc42 pathway and downstream PAK1/Cofilin cascade. The overexpression of SNPH increased mitochondrial fusion and deterred the liver metastasis ability of CRC cells in vivo. Together, our results suggest that SNPH suppression imposed by the HIF-1α/miRNA-130a-3p axis under hypoxia conditions promotes the liver metastasis of CRC cells by activating the AKT/cdc42-PAK1/Cofilin cascade through mitochondrial dynamics-mediated ROS production.

## Introduction

Colorectal cancer (CRC) is the third most common cancer worldwide [1]. Approximately 50% of patients develop liver metastasis during cancer progression, which causes severe deterioration of patient health and is a major reason for CRC-related death. Previous studies suggest that the process of CRC liver metastasis is highly complex and requires further investigation to deepen our understanding.

Mitochondria are vital organelles in cells, functioning as important sites for energy production, metabolism, calcium regulation and ROS generation. Mitochondria display dynamic morphology through continuous fission/fusion and are translocated to the leading edges of migrating cells, impacting cancer metastasis ability [2–5]. However, it is still unclear whether mitochondrial translocation affects the liver metastatic ability of cancer cells in CRC.

Syntaphilin (SNPH) is a membrane-associated protein that regulates mitochondrial dynamics. A previous study has shown that SNPH inhibits mitochondrial fission and movement [6]. In doing this, SNPH anchors mitochondria to the cytoskeletal microtubules, blocking their movement and allowing the mitochondria to supply energy locally to the cell edge. Caino et al. reported that SNPH could reduce the speed and distance of single mitochondrial movement and thus inhibit tumor metastasis. However, the effects of SNPH regulation on mitochondrial dynamics and cancer metastasis in colorectal cancer remain unclear for now [7]. It has also been reported that altering oxygen levels is pivotal for promoting mitochondrial dynamics. Hypoxia-inducible factor 1 (HIF-1) regulates several types of mitochondrial morphological changes, such as mitochondrial fission, fusion, and mitophagy [8]. Recent studies have found that SNPH is significantly down-regulated by hypoxia, but the specific regulatory mechanisms remain unknown [9].

The dynamics of the actin cytoskeleton regulate cell morphological changes, such as lamellipodia and filopodia formation, resulting in the directional migration and invasion of cancer cells [10]. The Rho GTPases regulate the formation of the cellular cytoskeleton by spatiotemporally controlling F-actin polymerization/depolymerization and the formation of actin-based protrusions and bundles [11]. However, the precise way in which SNPH can be integrated into actin cytoskeleton signaling pathways involved in cell metastasis is still unknown.

In the present study, we systematically investigated the effects of SNPH-mediated mitochondrial dynamics on the metastasis of CRC cells both in vitro and in vivo. In hypoxic conditions, HIF-1α transcriptionally activated miR-130a-3p, which targeted SNPH mRNA to inhibit its protein expression. SNPH downregulation activated the AKT/cdc42-PAK1/Cofilin cascade by promoting mitochondrial fission and ROS production in CRC cells, resulting in increased formation of filopodia and invasiveness of CRC cells. Our study unveils the mechanism of SNPH downregulation in the hypoxia microenvironment of CRC, and the underlying molecular mechanism of colorectal cancer liver metastasis was explored.

## Materials and methods

### Data acquisition and preprocessing

The DESeq2-normalized expression profiles of colorectal cancer samples and cancer-adjacent samples of TCGA were extracted from UCSC Toil RNAseq Recompute Compendium (ref: Toil enables reproducible, open source, big biomedical data analyses) (https://xenabrowser.net/datapages/?dataset=TCGA-GTEx-TARGET-gene-exp-counts.deseq2-normalized.log2&host=https%3A%2F%2Ftoil.xenahubs.net&removeHub=https%3A%2F%2Fxena.treehouse.gi.ucsc.edu%3A443). The expression data of the above TCGA-DEGs were extracted from the CRC datasets with liver metastasis from GSE6988 [12], GSE50760 [13], and GSE22834 [14]. The intersection of the DEGs between cancer and cancer-adjacent tissues, and the DEGs between primary cancer and liver-metastasis tissues of GEO datasets, were further intersected with mitochondria-related genes from MitoCarta 3.0 [15].

### Cell culture and tissue collection

The human colorectal cancer cell lines (HCT116, Caco2, RKO, SW480) and HEK293T cell line were purchased from the National Collection of Authenticated Cell Cultures (Shanghai, China), and cultured in RPMI-1640, MEM, Leibovitz’s L-15 or modified Eagle medium (DMEM) (Gibco, USA) at 37 °C in 5% CO_2_. High liver metastasis capacity cell lines (Caco2-H and RKO-H) were established by our research group previously [16]. All the media were supplemented with 10% FBS (BI, Israel) and 1% penicillin/streptomycin (Gibco, USA).

Fresh tissue and paraffin-embedded tissue from 117 CRC patients were collected from Liaoning Cancer Hospital & Institute (Shenyang, China). Representative regions containing viable tumor (>80% cellularity) were selected, excluding areas with necrosis or hemorrhage (defined as >10% involvement by H&E evaluation). Table S1 shows the clinicopathological features of patients with CRC. Approval to conduct studies with human participants was granted by the Ethics Committee of Liaoning Cancer Hospital & Institute (20201202). Informed consent was obtained from all patients.

### Total RNA extraction and real‑time quantitative polymerase chain reaction (RT‑qPCR)

The RNA of the tissues and cell lines was extracted using Trizol (Takara, Japan). cDNA synthesis was carried out using the PrimeScript^TM^ RT reagent Kit (Takara, Japan), and the Mir-X miRNA First-Strand Synthesis Kit (Takara, Japan) was used for miRNA reverse transcription. qPCR was performed using SYBR Green PCR Master Mix (Takara, Japan) on a CFX96 Real-Time PCR Detection System (Bio-Rad, USA), following the manufacturer’s instructions. Endogenous regulation was provided by GAPDH and U6 snRNA. Additional file: Table S3 presents the primer sequences utilized in this study.

### Immunohistochemistry, immunofluorescence, and western blotting assay

CRC tissues were processed for IHC and western blot assay as previously described [17]. Firstly, the slides were treated with SNPH, HIF-1α and Ki67 antibodies at 4 °C overnight, then incubated with HRP-conjugated secondary antibodies at 37 °C for 30 min. Finally, the sections were stained with DAB (Gene Tech, China) and counterstained with hematoxylin. Afterward, the images were captured using a microscope DMi8 (Leica, Germany).

Cells seeded on coverslips for more than 24 h were fixed in 4% formaldehyde for 30 min at room temperature, then permeabilized with 0.1% Triton X-100 for 15 min. After that, cells were treated with the cdc42^GTP^ primary antibody overnight at 4 °C, followed by incubation with species-specific secondary antibody for one hour at room temperature. Confocal images were captured using a ZEISS confocal microscope (Carl Zeiss, Germany) or an Olympus confocal microscope (Olympus Corporation, Japan).

For western blotting, the polyvinylidene fluoride membranes (Merck-Millipore, Germany) with transferred proteins were initially incubated with a primary antibody overnight at 4 °C, then treated with a secondary antibody conjugated to a horseradish peroxidase (HRP) tag. The band signals were then detected using a chemiluminescence imaging system (Bio-Rad, USA). The antibodies utilized in this study are presented in the Additional File: Table S4.

### Transwell assay

A 24-well transwell system (Corning, USA) was used to measure the migration ability of CRC cells. A total of 1–2 × 10^5^ tumor cells were seeded in the upper transwell chambers with serum-free medium, and the lower chambers were filled with 600 μL culture medium containing 10% FBS. After incubating at 37 °C for 48 h, cells were fixed with methanol, stained with 1% crystal violet from Sigma-Aldrich, USA, and counted using ImageJ software. The invasion assay was performed similarly, except that the chamber was pre-coated with diluted-matrigel from Corning, USA.

### Migration track assays

Cell migration was assayed under 10X phase microscopy of the CQ1 Benchtop High-Content Analysis System (YOKOGAWA, Japan). The cells were plated in a confocal dish (NEST, China) and allowed to migrate for 24 h. Images were captured every 30 min and then analyzed by ImageJ.

#### F-actin staining

F-actin fractions were stained by Actin-Tracker Red (Beyotime, C2205S) or Actin-Tracker Deep Red (Beyotime, C2210S) according to a previously described method [18]. Briefly, cell monolayers at 70% confluence were fixed at room temperature with 4% formaldehyde solution for 30 min. After washing 3 times with 0.1% Triton X-100 (PBS) for 5 min each time, the cells were incubated with Actin-Tracker Red/Deep Red diluted at a ratio of 1:50 at room temperature for 60 min away from light. After washing 3 times with 0.1% Triton X-100 (PBS) for 5 min each time, the samples were imaged using a confocal microscope (Olympus Corporation, Japan).

### RAC1/cdc42 pull-down assay

The activity of the RAC1/cdc42 protein was determined using the RAC1/cdc42 Activation Magnetic Beads Pulldown Assay kit (Millipore, Germany) according to the manufacturer’s instructions. Briefly, cell monolayers at 85-90% confluence were rinsed twice with ice-cold PBS and were lysed with 500 μL ice-cold lysis on ice. The lysates detached from plates with a cell scraper, as well as 10 µL RAC1/cdc42 Assay Reagent (PAK-1 PBD, Magnetic beads), were transferred to microfuge tubes on ice and incubated for 45 min at 4 °C with gentle agitation. The beads were pelleted by a magnetic tube stand, washed 3 times with lysis, and then resuspended with 40 µL of 2× loading buffer and boiled for 5 min. The suspension was isolated from beads by brief centrifugation and was detected by Western Blot with the antibodies of RAC1 and cdc42.

### Mitochondrial network imaging

MitoTracker® Red CMXRos (Invitrogen™, M7512) was utilized to monitor mitochondrial morphology in CRC cells. The cells were observed under the confocal microscope (Olympus FV 1000, Japan). To measure the length of mitochondria, the ImageJ software was employed.

### Electron microscopy

The liver metastasis tissues were fixed using glutaraldehyde, followed by post-fixation with OsO4 and dehydration with alcohol. Thin sections were stained with uranyl acetate and lead citrate. Images were acquired on a TEM (HITACHI, Japan).

### Luciferase reporter assay

A total of 2.5 × 10^4^ CRC were co-transfected with GV238-MIR130A-HG-promoter (Genechem, China) and siNC or siHIF-1α (Genepharma, China) and cultured in 5% CO_2_ for 12 h, followed by 1% O_2_ for 24 h. To assess the luciferase activity of the cell lysates, a Dual-Luciferase Reporter System (Promega, United States) was employed.

In a separate experiment, a total of 2.5 × 10^4^ CRC were co-transfected with miR-130a-3p mimic and pmirGLO-SNPH-MUT or pmirGLO-SNPH-WT (Genepharma, China). After a 24-h incubation period, the luciferase activity in the cell lysates was analyzed using the Dual-Luciferase Reporter System from Promega.

### Detection of mitochondrial reactive oxygen species

Mitochondrial superoxide levels were measured using MitoSOX™ Red (Thermo Fisher Scientific). Briefly, cells were incubated with 5 μM MitoSOX™ Red at 37 °C for 30 min, washed, and analyzed by flow cytometry.

### Intrasplenic inoculation of human CRC cells in mice

The animal studies and procedures were approved by the Institutional Review Board of China Medical University, adhering strictly to their guidelines. Six-week-old male BALB/c nude mice were anesthetized with tribromoethanol. The spleen was surgically exposed through a sterile operation, and 2 × 10^6^ cancer cells were precisely injected into it using a 30 G needle. The spleen was removed 3 min later. The abdominal wall and skin were then sutured using 5-0 polyglycolic acid sutures. After four weeks, the mice were humanely euthanized, and their livers were carefully extracted and photographed for further analysis.

### Statistical analysis

Where necessary, experiments were replicated three times, and the resulting data were expressed as the mean ± standard error of the mean (SEM). SPSS 17.0 software (SPSS, Chicago, IL) was used for all statistical analyses, with a significance level set at *P* < 0.05. Independent-sample *t*-tests were applied to compare data between two groups when deemed appropriate. The associations between measured variables were examined using either Pearson’s or Spearman’s rank correlation analyses. The Kaplan-Meier survival curve and log-rank test were employed to discern subgroups of patients with distinct overall survival (OS).

## Results

### SNPH downregulation correlates with liver metastases and poor prognosis in CRC

**Fig. 1:**
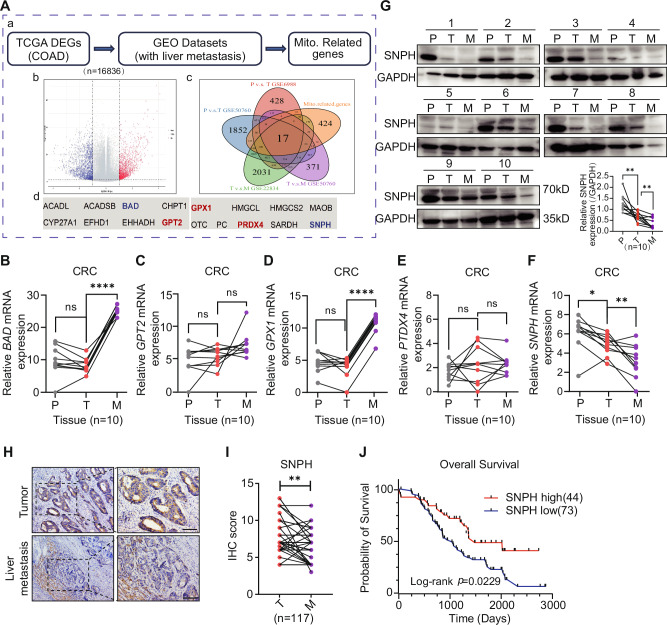
SNPH is progressively down-regulated in colorectal cancer and liver metastases and significantly contributes to poor patient prognosis. **A** (a) Datasets and bioinformatic analysis workflow. (b) Volcano plot showing 10029 downregulated and 6807 upregulated genes in CRC (*P* < 0.05) compared to peritumor (TCGA-COAD). (c) A Venn diagram showing differentially expressed genes within GSE6988, GSE50760, and GSE22834 datasets and Mito-related genes. (d) 17 candidate Mito-related genes involved in CRC liver metastasis were listed. Continuous upregulated (Red) or downregulated (Blue) genes in P → T → M were marked in addition. **B**–**F** The relative expression of *BAD*, *GPT2*, *GPX1*, *PRDX4* and *SNPH* was measured by RT-qPCR in paired tissues from CRC patients. **G** Western blot analyses for expression levels of SNPH in paired tissues from CRC patients. **H**, **I** Representative IHC staining images of SNPH in paired CRC tissues. Scale bars, 50 µM. **J** Kaplan–Meier curve analysis of overall survival in CRC patients by the expression of SNPH. P, peritumor; T, tumor; M, Liver metastasis. *P < 0.05; **P < 0.01; ***P < 0.001; ****P < 0.0001.

To identify candidate genes that influenced liver metastasis in CRC, we integrated expression data from TCGA and GEO datasets with primary tumor and liver metastases (LM) tissues, from which differentially expressed genes (DEGs) were analyzed. Next, we focused on genes involved in mitochondrial function and intersected the DEGs obtained from the analysis with the Mito3.0 dataset and identified 17 candidate genes (Fig. 1A). The trends of the expression levels of 5 (*BAD*, *GPT2*, *GPX1*, *PRDX4*, *SNPH*) out 17 genes were monotonically increasing or decreasing in the normal-cancer-metastasis sequence in the GSE50760 dataset (Fig. S1A–Q). Furthermore, RT-qPCR analysis for *BAD*, *GPT2*, *GPX1*, *PRDX4* and *SNPH* was performed. The results showed that *SNPH* was remarkably downregulated in primary tumors compared with peritumor tissues and even further decreased in LM compared with primary tumors (Fig. 1B-F and Fig. S2A–E). The differential expression of the other 4 genes could not be verified in the primary tumor and LM tissues. In addition, protein levels of SNPH were also observed by Western blots, indicating that SNPH protein levels in primary tumor tissues were lower than those in peritumor tissues, and were further decreased in paired LM tissues (Fig. 1G). The expression level analysis of SNPH mRNA from TCGA-COAD and GSE 22834 led to the same conclusion (Fig. S2F, G). We further verified the expression levels of SNPH in high liver metastatic ability CRC cell lines (Caco2-H and RKO-H) established previously [16]. Western blot results showed that the expression of SNPH was lower in Caco2-H and RKO-H than in their parental cells (Caco2 and RKO) (Fig. S2H). These results suggest that SNPH is decreased in CRC and further down-regulated in LM.

Immunohistochemical analysis in a cohort of 117 CRC patients with LM further confirmed that SNPH was decreased in LM tissues compared with peritumor tissues (Fig. 1H, I). Furthermore, we found that CRC patients with those exhibiting low SNPH expression demonstrated significantly poorer prognosis in comparison to those with high SNPH expression (Fig. 1J). Multivariable Cox regression determined that SNPH high expression and well/moderately pathological differentiation were independent predictive factors for a favorable prognosis in CRC patients (Table S2). Two cohorts, GSE71187 [19] and GSE17538 [20], were included in the prognostic analysis. In the GSE71187 cohort, low SNPH expression showed worse OS (*P* = 0.0385) (Fig. S2I). In the GSE17538 cohort, while a low expression of SNPH did not exhibit a significant correlation with disease-free survival (DFS) (*P* = 0.093), a decrease in SNPH expression was found to be associated with poorer OS (*P* = 0.0044) (Fig. S2J, K). Therefore, our findings suggest that SNPH expression is closely related to the development of CRC.

### SNPH downregulation promotes migration of CRC cells by remolding RAC1/cdc42-mediated filopodia formation

**Fig. 2:**
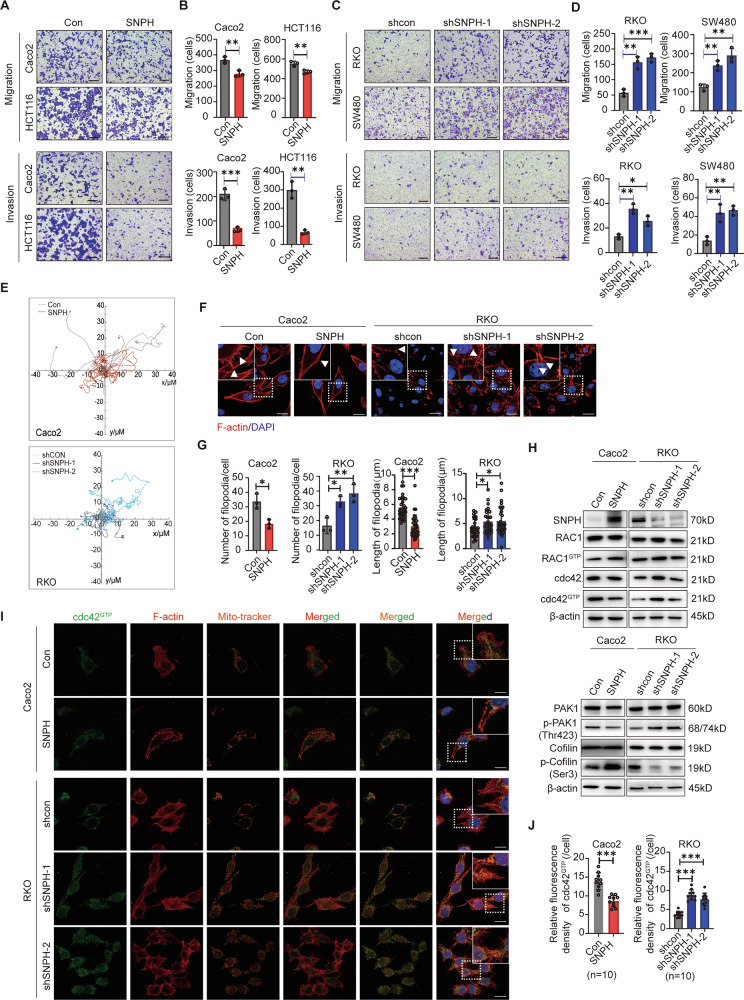
SNPH downregulation promoted metastasis of CRC cells by affecting Rac1/cdc42-mediated lamellipodia formation. **A**–**D** Transwell migration and invasion analysis for SNPH stably overexpressed or knockdown CRC cells. **E** Migration tracks of individual cells in Caco2 and RKO, as indicated, were shown as a spider plot. **F**, **G** Phalloidin staining in CRC cells as indicated. Triangles indicate lamellipodia formation. Scale bars, 5 µM. **H** Western blot analyses for SNPH, RAC1, RAC1^GTP^, cdc42, cdc42^GTP^, PAK1, p-PAK1(Thr423), Cofilin, p-Cofilin (Ser3), β-actin expression in CRC cells with treatment as indicated. **I**, **J** Representative images of IF staining for cdc42^GTP^ (green), F-actin (red), mitochondria (Mito-tracker-Deep-Red, orange) in CRC cells with different treatments as indicated. Scale bars, 5 µM. *P < 0.05; **P < 0.01; ***P < 0.001.

To explore the effects of SNPH-regulated mitochondrial dynamic changes on CRC cells, we performed proliferation, apoptosis, cell cycle, migration, and invasion assays with CRC cell lines. CRC cells with low (Caco2, HCT116) or high (RKO, SW480) SNPH expression were selected for the establishment of SNPH overexpression or knockdown cell models, respectively (Fig. S3A–C). SNPH and Mito-Tracker Red staining revealed that, in comparison to control cells, Caco2 cells exhibiting SNPH overexpression displayed significant alterations in their mitochondrial elements. Specifically, these mitochondria became significantly elongated, interconnected, and aggregated towards the nucleus (Fig. S3D, E). In contrast, the percentages of fragmented mitochondria were conspicuously elevated in RKO cells following the knockdown of SNPH (Fig. S3D, E). Analysis via western blot revealed that SNPH knockdown significantly elevated levels of p-Drp1 in RKO cells, while SNPH overexpression exhibited the opposite effect in Caco2 cells (Fig. S3F). As shown in Fig. S4A–C, SNPH expression alteration showed no significant effects on cell proliferation or cell apoptosis in CRC cells. Quantitative analysis by flow cytometry indicated that SNPH overexpression and knockdown significantly decreased the percentage of CRC cells in G1 phase of cell cycle (Fig. S4D), which needs further exploration. However, cell migration and invasion were significantly decreased in CRC cells with SNPH overexpression, but were remarkably increased in cells with SNPH knockdown (Fig. 2A–D). Western blot analysis showed that, except for Snail, the expression of EMT markers was consistent among CRC cells with different SNPH expression levels (Fig. S4E). Migration track assays indicated that SNPH knockdown led to a greater range of movement of CRC cells, whereas SNPH overexpression restricted cancer cell movement (Fig. 2E). F-actin staining showed a remarkable alteration in the filopodial protrusion in the CRC cells with different SNPH expression levels. The protrusions were fewer and shorter in Caco2 cells with SNPH overexpression, while the protrusions were increased and elongated in RKO cells with SNPH knockdown (Fig. 2F, G).

The small Rho GTPases are pivotal in regulating actin cycling and cytoskeletal reorganization. We examined the expression of two key regulators of cell protrusions, RAC1 and cdc42. Western blotting results showed no significant alteration in their expression levels in CRC cell lines (Fig. 2H). Pulldown of GTP-bound RAC1 demonstrated minor changes among different cell lines, whereas the activated form of cdc42 (cdc42^GTP^) exhibited significant changes in both cell lines with varying SNPH expression (Fig. 2H). Meanwhile, western blotting analysis revealed that when SNPH was knocked down, the cdc42 downstream pathway PAK1-Cofilin was activated, leading to an upregulation of p-PAK1 and a downregulation of p-Cofilin. Conversely, the opposite results were observed when SNPH was overexpressed. Immunofluorescence co-localization studies of cdc42^GTP^, mitochondria and F-actin in SNPH-manipulated CRC cells have been performed. As shown in Fig. 2I, J, SNPH overexpression reduces activated cdc42 expression, inhibits the aggregation of activated cdc42 to the cell membrane, diminishes the co-localization of cdc42 with F-actin, and decreases mitochondrial accumulation at the cell cytoskeleton. Conversely, the opposite results were observed when SNPH was knocked down. These cumulative data suggest that reduced expression of SNPH promoted filopodia formation, likely by increasing the activation of the small Rho GTPase cdc42.

### Hypoxia-induced SNPH down-regulation promotes filopodia formation and metastasis of CRC cells

**Fig. 3:**
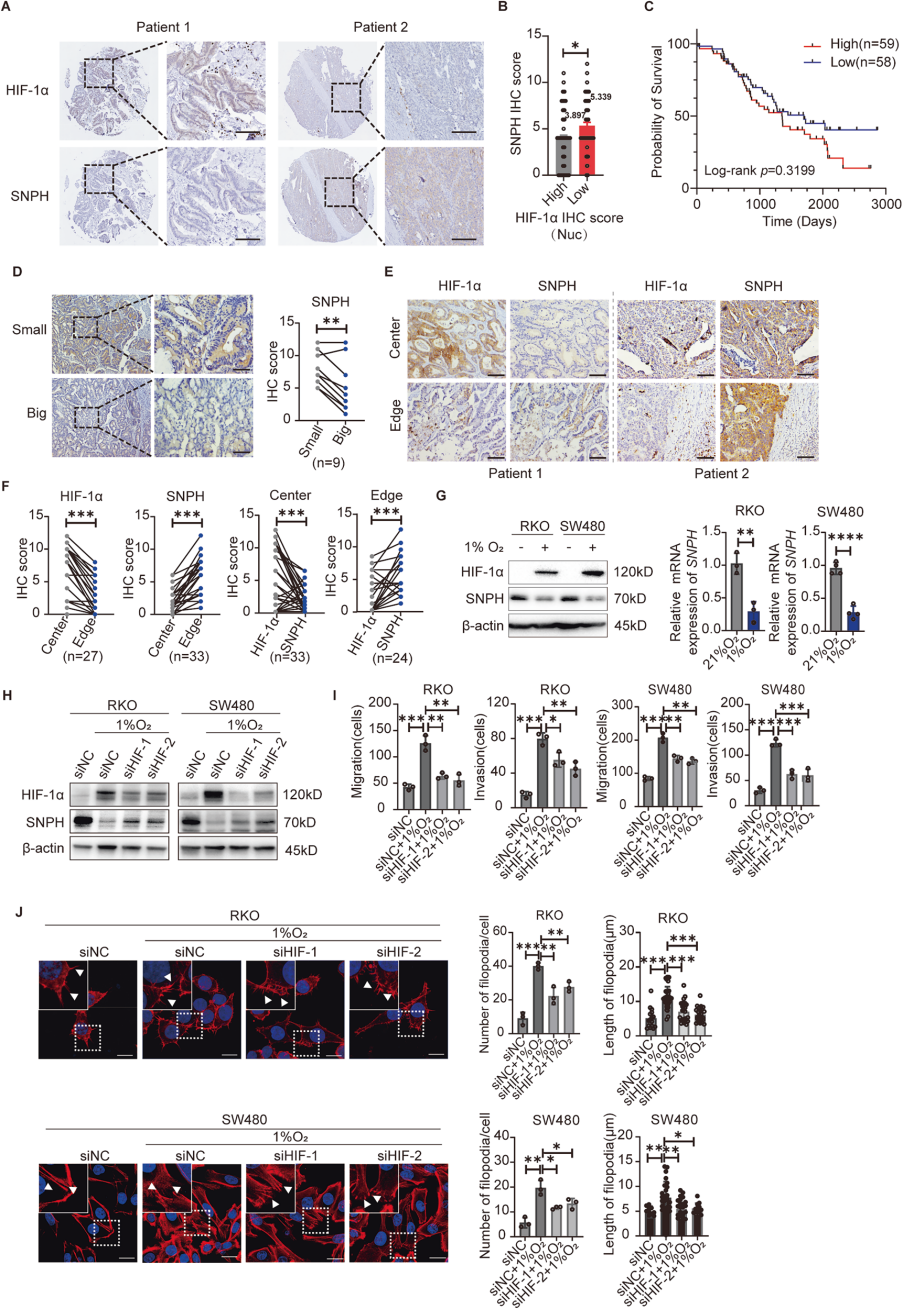
Hypoxia-mediated SNPH down-regulation in CRC. **A** IHC analysis of CRC samples showed a negative correlation between HIF-1α and SNPH. Scale bars, 50 µM. **B** SNPH IHC score in different HIF-1α IHC score (Nuc) samples in CRC. **C** Kaplan–Meier curve analysis of overall survival in CRC patients by the expression of HIF-1α (Nuc). **D** Relative expression of SNPH in liver metastasis lesions of different diameters in the same patient with CRC. Scale bars, 50 µM. **E**, **F** Representative IHC staining images of SNPH and HIF-1α in the edge and center of the same tumor. Scale bars, 50 µM. **G** Western blot and RT-qPCR analyses of the SNPH expression in CRC cells cultured under normoxia or hypoxia conditions. **H** Western blot analyses the expression of SNPH in CRC cells transfected with HIF-1α siRNAs or siNC under normoxia or hypoxia conditions. **I** Transwell migration and invasion analysis for CRC cells with treatment as indicated. **J** Phalloidin staining in CRC cells as indicated. Triangles indicate lamellipodia formation. Scale bars, 5 µM.*, *P* < 0.05; **, *P* < 0.01; ***, *P* < 0.001.

Next, we embarked on a quest to unravel the molecular mechanism underlying SNPH downregulation in CRC cells. The oxygen content in the tumor microenvironment has been shown to affect the dynamics of mitochondria [8]. Previous studies embarked on a quest to unravel also suggest that SNPH can be significantly down-regulated under hypoxic conditions [9]. Therefore, we assessed the expression of SNPH and HIF-1α in primary tumor tissues obtained from CRC patients exhibiting LM. Immunohistochemical analysis showed a reverse correlation between HIF-1α and SNPH expression (Fig. 3A). CRC patients with high HIF-1α (Nuc) scores showed relatively lower SNPH expression and exhibited a poorer overall survival rate in comparison to those with low HIF-1α (Nuc) scores, although not statistically significant (Fig. 3B, C). It is suggested that the degree of hypoxia in the tumor microenvironment correlates with tumor diameters; we hence assessed the expression of SNPH in liver metastases with different diameters. The IHC results showed weaker SNPH staining in bigger liver metastatic lesions as compared to smaller metastatic lesions (Fig. 3D). IHC assays of SNPH and HIF-1α in the same liver metastatic lesions indicated lower expression of SNPH but higher expression of HIF-1α in the center of lesions, while opposite results were observed in the edges of tumors (Fig. 3E, F).

**Fig. 4:**
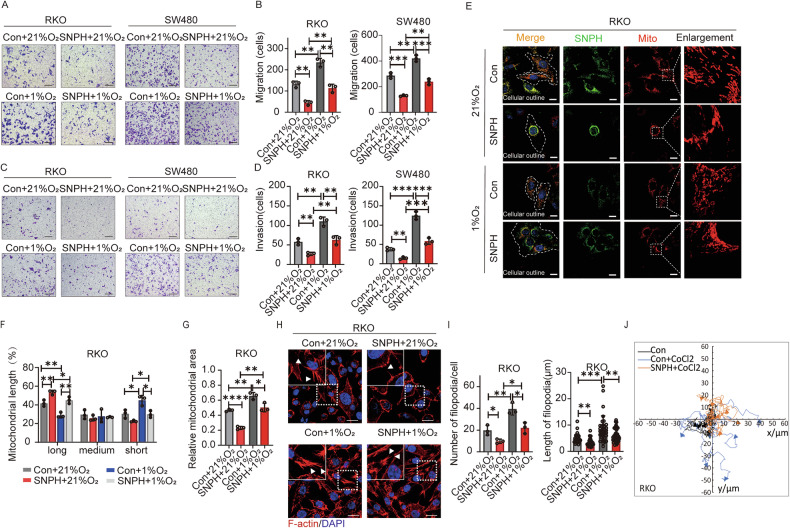
Hypoxia-mediated SNPH down-regulation promotes lamellipodia formation and metastasis of CRC cells. **A**–**D** Transwell migration and invasion analysis for stably transfected CRC cells with treatment as indicated. **E** Representative images of IF staining for SNPH (green) and mitochondria (Mito-tracker-red) in CRC cells with different treatments as indicated. Scale bars, 5 µM. **F**, **G** Average length and area of mitochondria per cell were analyzed in CRC cells with treatment as indicated in (**E**). **H**, **I** Phalloidin staining in CRC cells as indicated. Triangles indicate lamellipodia formation. Scale bars, 5 µM. **J** Migration tracks of individual cells in RKO, as indicated, were shown as a spider plot. *P < 0.05; **P < 0.01; ***P < 0.001.

IHC results indicate that SNPH and HIF-1α expression levels are negatively correlated. To further investigate their regulatory relationship, we examined the protein and RNA levels of HIF-1α in CRC cells with varying SNPH expression levels. The results showed no significant differences between the groups (Fig. S5A). Next, we assessed the changes in SNPH expression levels under hypoxic conditions in CRC cells. Our results showed that both protein and mRNA levels of SNPH were significantly down-regulated under 1% oxygen conditions, in a time-dependent manner (Fig. 3G and S5B, C). After knocking down HIF-1α with siRNA, the down-regulation effects of hypoxia on SNPH expression were partially suppressed in CRC cells (Fig. 3H). Cell migration, invasion, and F-actin staining results were consistent (Fig. 3I, J and S5E). We next investigated the impact of hypoxia-mediated SNPH downregulation on CRC cell migration. Our results show that both cell migration and invasion were enhanced by 1% O_2_ treatment, and were partially reversed by SNPH overexpression in both Caco2 and SW480 cells (Fig. 4A–D). The immunofluorescence data showed that 1% O_2_ treatment induced the downregulation of SNPH. Mito-tracker red staining showed that 1% O_2_ treatment promoted mitochondrial fission and dispersion to the edges of CRC cells, which were partially suppressed by SNPH overexpression (Fig. 4E–G). F-actin staining and migration tracks experiments indicate that hypoxia condition promoted the formation of filopodia and increased cell movement ranges, whereas SNPH overexpression partially suppressed these phenotypes (Fig. 4H–J). Collectively, our data indicate that HIF-1α suppressed SNPH expression in the hypoxia microenvironment and promoted cell migration in CRC.

### The HIF-1α/miR-130A-3p axis represses SNPH expression and promotes filopodia formation

**Fig. 5:**
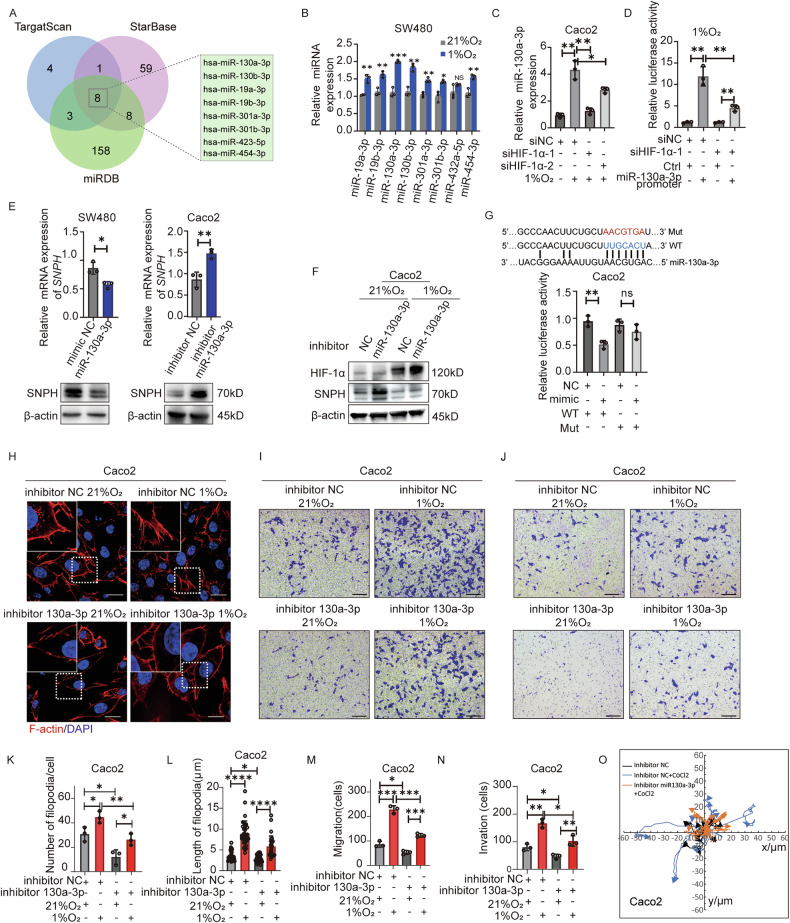
HIF-1α/miR-130A-3p impresses SNPH expression and promotes the lamellipodia formation and metastasis of CRC cells. **A** A Venn diagram showing candidate miRNAs that may regulate SNPH mRNA expression. **B** qRT-PCR analyses the expression of miRNAs in CRC cells cultured under normoxia and hypoxia conditions. **C** qRT-PCR analyses the expression of miR-130a-3p in Caco2 after transfected with HIF-1a siRNAs or siNC under normoxia or hypoxia conditions. **D** The luciferase activities of 293 T after co-transfected with luciferase reporter vectors GV238-miR-130A-3P-HG-promoter and siNC or siHIF-1α were examined. **E** qRT-PCR and western blot analyze the expression of SNPH in SW480 and Caco2 after transfected with miR-130a-3p mimic or mimic NC and miR-130a-3p inhibiter or inhibiter NC. **F** Western blot analyses the expression HIF-1α and SNPH in Caco2 after transfected with miR-130a-3p inhibiter or inhibiter NC under normoxia and hypoxia conditions. **G** The luciferase activities of Caco2 after co-transfected with luciferase reporter vectors miR-130a-3p mimic and pmirGLO-SNPH-MUT or pmirGLO-SNPH-WT were examined. **H**, **K**, **L** Phalloidin staining in Caco2 as indicated. Triangles indicate lamellipodia formation. Scale bars, 5 µM. **I**, **J**, **M**, **N** Transwell migration and invasion analysis for Caco2 with treatment as indicated. **O** Migration tracks of individual cells in Caco2 as indicated were shown as spider plot demonstrates. *P <0.05; **P < 0.01; ***P < 0.001; ****P < 0.0001.

Driven by the desire to unravel the mechanism governing hypoxia-modulated SNPH expression, we examined the transcriptional regulation of SNPH. The prediction of UCSC (https://genome.ucsc.edu/) and JASPAR (https://jaspar2020.genereg.net/) databases revealed that the binding site of HIF-1α was not included in the promoter region of SNPH. Retrieval of public ChIP Seq data shows that the mRNAs bound by HIF-1α do not contain sequences related to the transcriptional regulatory region of SNPH [21, 22]. Furthermore, the introduction of HIF-1α siRNA and overexpression vector led to no change in the luciferase activity downstream of the SNPH-promoter (Fig. S5D). These results indicated that HIF-1α may regulate SNPH expression through an indirect pathway. Previous studies reported that HIF-1α could repress the expression of target genes through transcriptional activation of microRNAs [23, 24]. Next, we searched for microRNAs that might target SNPH through public database analysis. We used three online bioinformatics tools, miRDB (https://www.mirdb.org/), TargetScan (https://www.targetscan.org/vert_80/), and ENCORI (https://starbase.sysu.edu.cn/index.php) to predict potential target miRNAs for SNPH. Figure 5A illustrates that 8 potential miRNA, i.e., miR-19a-3p, miR-19b-3p, miR-130a-3p, miR-130b-3p, miR-301a-3p, miR-301b-3p, miR-423a-5p, miR-454a-3p were common in the predictions using the three databases. To identify the miRNAs that were primarily governed by HIF-1α, we conducted an RT-qPCR analysis of the expression levels of 8 predicted miRNAs. Amongst these 8 miRNAs, miR-130a-3p exhibited the most notable increase in expression (Fig. 5B). Utilizing the ENCORI online data analysis platform (https://starbase.sysu.edu.cn/index.php), we observed that miR-130a-3p is abundantly expressed in CRC and correlates with an unfavorable prognosis for CRC patients (Fig. S6A, B). Transcriptional regulatory regions of miR-130a-3p were selected for transcription factor prediction using the UCSC website. Then the Jaspar online tool was used to score candidate transcription factors, from which we identified HIF-1α as a potential target regulating miR-130a-3p (Fig. S6C). HIF-1α silencing in CRC cells cultured in 1% O_2_ conditions showed partial restoration of miRNA-130a-p expression (Fig. 5C). Furthermore, the introduction of HIF-1α siRNA led to a marked decrease in the luciferase activity downstream of the miRNA-130a-3p-HG-promoter (Fig. 5D). This finding implies that HIF-1α transcriptionally modulates the expression levels of miR-130a-3p.

Western blot and RT-qPCR analysis showed that miR-130a-3p mimics led to a notable decrease in SNPH expression levels, whereas miR-130a-3p inhibitors resulted in a significant increase (as seen in Fig. S6D and Fig. 5E). The inhibitory effects of hypoxia on SNPH expression were partially restored by miR-130a-3p inhibitors (Fig. 5F). Additionally, miR-130a-3p mimics led to a marked decrease in the luciferase activity associated with the wild-type SNPH 3’UTR region, whereas no such reduction was observed in the mutant 3’UTR (Fig. 5G). RNAscope analysis of colorectal cancer tissues revealed co-localization of miRNA-130a-3p and SNPH in the cell cytoplasm (Fig. S6E). Furthermore, upon transfection with miR-130a-3p inhibitors, the formation of cell filopodia, as well as the migration and invasion capabilities of Caco2 cells cultured under 1% O_2_ conditions, were notably reduced, along with a decrease in migration tracks (Fig. 5H–O). Taken together, our data suggest that inhibited SNPH expression by up-regulated miRNA-130a-3p levels in the hypoxic microenvironment promoted CRC cell migration and invasion.

### SNPH regulates filopodia formation and metastasis of CRC cells through ROS-stimulated cdc42-PAK1-Cofilin pathway

**Fig. 6:**
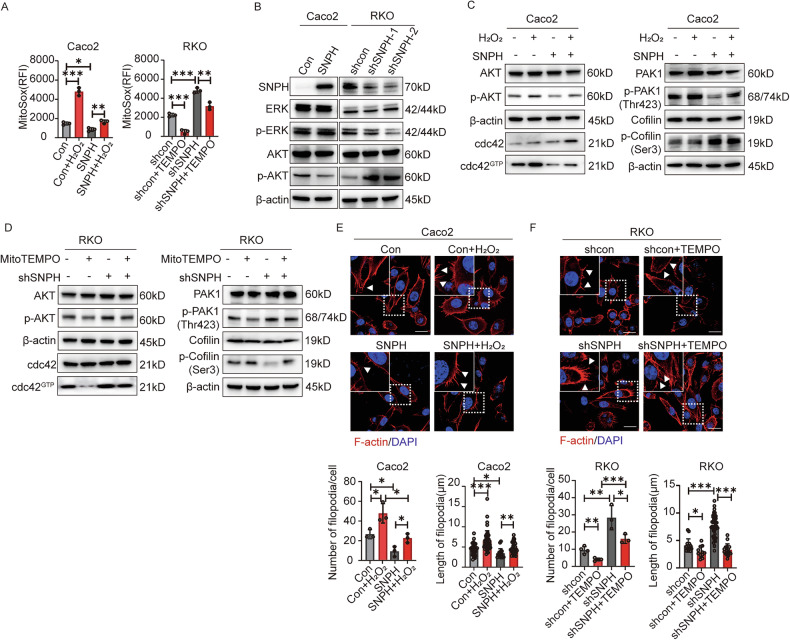
SNPH regulates lamellipodia formation and metastasis of CRC cells through ROS-modulated activity of the cdc42-PAK1-Cofilin pathway. **A** The intracellular ROS levels in CRC cells treated with H_2_O_2_ or Mito-TEMPO were assessed using flow cytometry. **B** Western blot analyses were performed to determine the protein levels of AKT, p-AKT, ERK, and p-ERK in CRC cells treated as indicated. **C** Western blot analyses for protein levels of AKT, p-AKT, cdc42, cdc42^GTP^, PAK1, p-PAK1 (Thr423), Cofilin, p-Cofilin (Ser3) in SNPH overexpressed Caco2 treated with or without H_2_O_2_. **D** Western blot analyses for protein levels of AKT, p-AKT, cdc42, cdc42^GTP^, PAK1, p-PAK1 (Thr423), Cofilin, p-Cofilin (Ser3) in SNPH knockdown RKO treated with or without Mito-TEMPO. **E**, **F** Phalloidin staining in Caco2 and RKO as indicated. Triangles indicate lamellipodia formation. Scale bars, 5 µM. *P < 0.05; **P < 0.01; ***P < 0.001.

Mitochondria serve as the principal generator of reactive oxygen species (ROS) within cells, and these ROS function as secondary messengers, playing a crucial role in regulating numerous signaling pathways, including ERK and AKT. The subcellular positioning and dynamic processes of fission/fusion in mitochondria are integral to maintaining ROS levels at the subcellular level [25]. Recent studies have uncovered that hypoxia stimulates reactive oxygen species (ROS) generation [26]. Therefore, we surmised that alterations in ROS production and redistribution imposed by hypoxia mediated SNPH-regulated mitochondrial dynamics might be involved in the regulation of ERK or AKT activities in CRC cells. Flow cytometry analysis showed that hypoxia promoted ROS generation, which can be partially reversed by SNPH overexpression (Fig. S7B). The reduction in SNPH expression significantly enhanced the generation of ROS and was notably mitigated when the ROS scavenger MitoTEMPO was administered (Fig. 6A and S7A). Analysis via Western blot revealed that SNPH knockdown significantly elevated levels of p-AKT in RKO cells, while SNPH overexpression exhibited the opposite effect in Caco2 cells, although different SNPH expression showed no effects on p-ERK expression (Fig. 6B). To strengthen our understanding of ROS’s pivotal role in AKT activation and downstream signaling cascades, we modulated ROS levels in CRC cells using H_2_O_2_ and MitoTEMPO treatments. Western blot analysis subsequently revealed that H_2_O_2_ exposure led to a notable elevation in the protein abundance of p-AKT, cdc42^GTP^, and p-PAK1, while concomitantly reducing the expression of p-Cofilin. Conversely, ROS scavenging via MitoTEMPO application displayed inverse effects (Fig. 6C, D). F-actin staining showed that H_2_O_2_ promoted filopodia formation, which could be partially restored by SNPH overexpression (Fig. 6E). Conversely, upon treatment with MitoTEMPO, an opposite trend was observed in filopodia formation (Fig. 6F). In summary, our research suggests that enhancing mitochondrial dynamics via downregulation of SNPH triggers the PAK1-Cofilin signaling pathway through ROS-mediated activation of the AKT-cdc42^GTP^ cascade.

### SNPH up-regulation inhibited liver metastasis of CRC cells in vivo

**Fig. 7:**
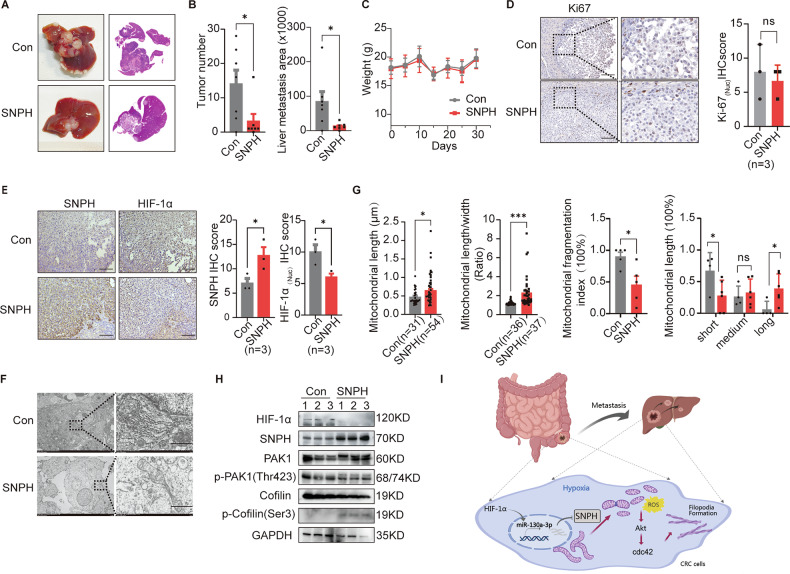
SNPH up-regulation inhibited liver metastasis of CRC cells in vivo. **A**, **B** Representative liver tissues from various experimental groups were examined to demonstrate the liver metastasis potential in nude mouse tumor models. H&E staining in Liver metastatic nodules. **C** Mouse weights curve. **D** IHC analysis of Ki67 in liver metastatic nodules of nude mice treated as indicated. Scale bars, 50 µM. **E** IHC analysis of SNPH and HIF-1α in liver metastatic nodules of nude mice treated as indicated. Scale bars, 50 µM. **F**, **G** Representative TEM (Transmission Electron Microscopy) images showcasing the mitochondrial network within liver metastatic nodules of nude mice, following the indicated treatment. Scale bar: 10 μM. **H** Western blot analyses for protein levels of SNPH, HIF-1α, PAK1, p-PAK1(Thr423), Cofilin, and p-Cofilin (Ser3) in liver metastatic nodules of nude mice treated as indicated. *P<0.05. **I** Schematic depicting the downregulation of SNPH by the HIF-1α/miR130a-3p axis in hypoxia condition. Cytoskeleton remodeling regulated by SNPH downregulation mediated by mitochondrial dynamics promotes the liver metastasis of CRC via ROS-mediated AKT/cdc42 pathway activity.

To investigate the influence of SNPH expression on CRC cell metastasis to the liver in vivo, an orthotopic nude mice model of CRC LM was developed using CRC cells with varying degrees of SNPH expression. As depicted in Fig. 7A, B, mice inoculated with CRC cells overexpressing SNPH displayed significantly reduced liver metastasis lesions in terms of both size and number compared to the control group, with Ki-67 staining and the weights of mice in the two groups being similar (Fig. 7C, D). IHC analysis indicated that there was a negative correlation between IHC scores of SNPH and HIF-1α expression (Fig. 7E). Utilizing transmission electron microscopy (TEM), we analyzed mitochondrial morphology in nude mouse models of CRC LM tissues to elucidate changes in mitochondrial dynamics. Our findings revealed significantly decreased mitochondrial length on average in control LM tissues than that in SNPH LM tissues (Fig. 7F, G). Western blot was performed to detect HIF-1α, SNPH, PAK1, p-PAK1, Cofilin and p-Cofilin protein expression levels in LM nodules of nude mice with treatment as indicated. The results showed that, in the LM nodules of SNPH overexpression nude mice, there was an elevation in the protein levels of SNPH and p-Cofilin, while the expression of HIF-1α and p-PAK1 was reduced (Fig. 7H). Therefore, our results showed that SNPH overexpression repressed CRC LM in vivo, which was associated with a hypoxic microenvironment.

## Discussion

To explore the molecular mechanisms of CRC liver metastasis, public CRC datasets containing liver metastases were selected for analysis, and 17 potential genes were identified in this study. However, changes in the expression of these 17 genes were not consistent in the order of peritumor → tumor → liver metastasis. The expression levels of 12 genes (*ACADL*, *ACADSB*, *CHPT1*, *CYP27A1*, *EFHD1*, *EHHADH*, *HMGCL*, *HMGCS2*, *MAOB*, *OTC*, *PC*, *SARDH*) were downregulated in tumors compared with peritumor tissues, but significantly upregulated in liver metastasis. It suggests that these genes’ roles in the tumor initiation and metastasis process might be different. RT-qPCR and western blot verification in CRC tissues showed that, among the 5 genes selected, the expression variation of *SNPH* was most significant and consistent with previous data analysis. The tissue verification results of the other 4 genes were complicated; some were not even significant, possibly due to our small sample size.

Recent studies found that SNPH can inhibit tumor metastasis by inhibiting the aggregation of Drp1 to mitochondria or inhibiting the phosphorylation of Drp1, the speed of mitochondrial division and fusion, and the movement to the cortical cytoskeleton [7, 9, 27, 28]. Chen et al. discovered that radioresistant (RR) ESCC cells exhibited a sparse mitochondrial network and reduced SNPH levels. Notably, the expression of SNPH was correlated with the efficacy of radiotherapy and emerged as an independent predictor of survival among ESCC patients [29]. Fu et al. observed reduced SNPH levels in pathologically activated neutrophils (PMN) in cancer. Furthermore, the knockout of SNPH enhanced the spontaneous migration of PMN and augmented metastasis in mice [30]. Our findings in CRC demonstrate that the suppression of SNPH results in an augmentation of mitochondrial fragmentation and its relocation towards the cell periphery, thereby confirming the pivotal role of SNPH in regulating mitochondrial dynamics. Furthermore, suppressing the expression of SNPH promoted cell filopodia formation, migration and invasion, suggesting that low SNPH expression facilitated liver metastasis in CRC cells and contributed to an unfavorable prognosis for patients. However, our study revealed that the expression level of SNPH has no significant effect on cell proliferation, apoptosis, or cell cycle of CRC cells, which differs from the findings in other types of tumors [9, 29]. This suggests that the role of SNPH in different tumors may be varied. In addition, Flou-4 Flow cytometry analysis, lipid staining, and ATP generation analysis suggested that overexpression of SNPH can downregulate intracellular calcium levels, inhibit lipid synthesis, and inhibit ATP generation. The opposite results were obtained in SNPH knocked-down CRC cells, indicating that SNPH may regulate the biological behavior of CRC through multiple mechanisms, which requires further exploration (Fig. S7C–E).

Filopodia are actin-rich plasma membrane protrusions. Migration is the most characteristic physiological function of filopodia formation [31]. Our current research highlights that SNPH downregulation encouraged filopodia formation, along with enhancing cell migration and invasion in CRC cells. The Rho GTPases exhibit profound effects on cell morphology and actin cytoskeleton organization, wherein RAC1 stimulates the formation of F-actin-rich lamellipodia [32], whereas cdc42 triggers the extension of bundled F-actin projections known as filopodia [33]. In the present study, we used GST-pull-down assays to detect the activation of cdc42/RAC1. Our results showed that cdc42 was significantly more activated by SNPH downregulation, while Rac1 activation remained unchanged, which was consistent with the reported mechanisms of filopodia formation.

There are relatively few studies on the upstream regulatory mechanisms of SNPH expression. In our study, we found that SNPH was downregulated in hypoxic CRC cells, and its low expression correlated with high HIF-1α expression (Nuc) in the cancer samples of CRC patients. While our study identifies an inverse correlation between HIF-1α and SNPH, a key limitation is that HIF-1α IHC alone cannot definitively establish hypoxia as the causative factor, due to potential non-hypoxic stabilization of the protein. In subsequent studies, we would benefit from employing more specific hypoxia assessment techniques, such as pimonidazole staining or analysis of markers like CA-IX and GLUT-1, to conclusively link the hypoxic tumor microenvironment to the downregulation of SNPH.

JASPAR database predicted HIF-1 binding sites within the promoter region of the *miRNA-130a-3p* gene. Our data confirmed that miRNA-130a-3p and SNPH were spatially co-localized, and elevated miRNA-130a-3p levels down-regulated SNPH expression. Of note, other mechanisms may be underlying HIF-1α mediated inhibitory regulation of SNPH expression. In addition to hypoxia, there might be other possible mechanisms regulating SNPH expression, which need to be further investigated.

Prior research has indicated that miR-130a-3p plays a significant role in the development and progression of various cancers, encompassing HCC, cervical and ovarian cancers, glioblastoma, and prostate cancer [34], including CRC [35, 36]. The target genes of miR-130a-3p include *NF-kB, TNF-a, Smad4, TGF-b, PTEN, XIAP, Met, RUNX3, RAB5A, NRP1*, etc., which can regulate multiple signaling pathways in cancer cells. While the specific roles of miR-130a-3p in facilitating liver metastasis of CRC have remained elusive. Our results suggest for the first time that miR-130a-3p promotes liver metastasis in CRC by inhibiting the expression of SNPH.

With in vivo experiments, we observed an interesting phenomenon that mice belonging to the SNPH overexpression group displayed significantly reduced expression levels of HIF-1α in their liver metastases compared to the control group (Fig. 7H). The seemingly discrepant findings between our clinical observations and interventional animal experiments actually shed light on a more sophisticated regulatory relationship between SNPH and HIF-1α. Initially, the inverse correlation between HIF-1α and SNPH expression in human colorectal cancer samples suggested, but could not prove, that hypoxia drives SNPH downregulation. Our subsequent in vivo functional study, demonstrating that SNPH overexpression leads to reduced HIF-1α levels in liver metastases, provides strong causal evidence supporting this notion. This confirms that HIF-1α is indeed upstream of SNPH, acting as a negative regulator. The absence of an effect in cultured cells under standard conditions further strengthens the argument that this regulatory relationship is context-dependent, requiring the full complexity of the in vivo microenvironment, such as authentic hypoxia, to manifest. Most importantly, these collective findings allow us to propose a novel positive feedback loop model within tumors: Pathological hypoxia (e.g., in aggressive tumors or metastases) leads to HIF-1α accumulation, which transcriptionally represses SNPH expression. The subsequent loss of SNPH, a key regulator of mitochondrial anchoring, disrupts mitochondrial function and distribution. This alteration in mitochondrial dynamics may, in turn, promote a more glycolytic metabolism (Warburg effect) and/or increase mitochondrial reactive oxygen species (mtROS) production, both of which are known to further stabilize HIF-1α protein, thereby amplifying and sustaining the hypoxic response even under mild oxygen fluctuations. Therefore, what might appear as a contradiction is actually consistent with a model where HIF-1α acts as the initiator of SNPH downregulation, and SNPH downregulation then functions as an amplifier of the HIF-1α/hypoxic response, creating a feed-forward loop that potentiates tumor adaptation and aggressiveness.

In summary, our research reveals a common downregulation of SNPH in CRC tissues and their liver metastasis, leading to mitochondrial fission and translocation to cell edges, which contribute to the poor prognosis of patients. HIF-1α increases miRNA-130a-3p transcription, leading to decreased SNPH expression. Low expression of SNPH mediates mitochondrial fission, critically regulating liver metastasis in CRC cells by promoting filopodia formation. This process is mediated through ROS-stimulated AKT phosphorylation and subsequent activation of the cdc42/PAK1/Cofilin-1 pathway (Fig. 7I).

## Supplementary information

Supplementary File

Supplementary Material

## Data Availability

The data and materials analyzed in this study are accessible from the corresponding author upon reasonable request.
