# Supplementary material for: HIF-1α suppresses SNPH expression to facilitate liver metastasis of colorectal cancer through regulating mitochondrial dynamics and filopodia formation
Source: Cell Death Dis. 2026 Mar 26;17(1):380. doi: 10.1038/s41419-026-08551-1 (PMC13039215; doi:10.1038/s41419-026-08551-1)
Supplement: Supplementary file 1 — Supplementary File [file 41419_2026_8551_MOESM1_ESM.docx]

**HIF-1α suppresses SNPH expression to facilitate liver metastasis of colorectal cancer through regulating mitochondrial dynamics and filopodia formation**

Lei Zhan^1^, Xiaoxi Li^2^, Xiaoyan Li^3^, Qian Fei^4^, Yue Jin^1^, Jiaxing Yu^1^, Luyao Tian^1^, Feifei Li^1^, Chunning Li^2^, Qian Dong^1^, Yong Zhang^3^, Shulan Sun^2^, Jingdong Zhang^1,5*^

1. Medical Oncology Department of Gastrointestinal Cancer, Cancer Hospital of China Medical University, Liaoning Cancer Hospital & Institute, Cancer Hospital of Dalian University of Technology, Shenyang, Liaoning, 110042, China
2. Central Laboratory, Cancer Hospital of China Medical University, Liaoning Cancer Hospital & Institute, Cancer Hospital of Dalian University of Technology, Shenyang, 110042, China
3. Department of Pathology, Cancer Hospital of China Medical University, Liaoning Cancer Hospital & Institute, Cancer Hospital of Dalian University of Technology, Shenyang, Liaoning, 110042, China
4. Department of Oncology, Shengjing Hospital of China Medical University, Shenyang, Liaoning, 110042, China
5. Liaoning Key Laboratory of Gastrointestinal Cancer Translational Research, Shenyang, Liaoning, 110042, China

**
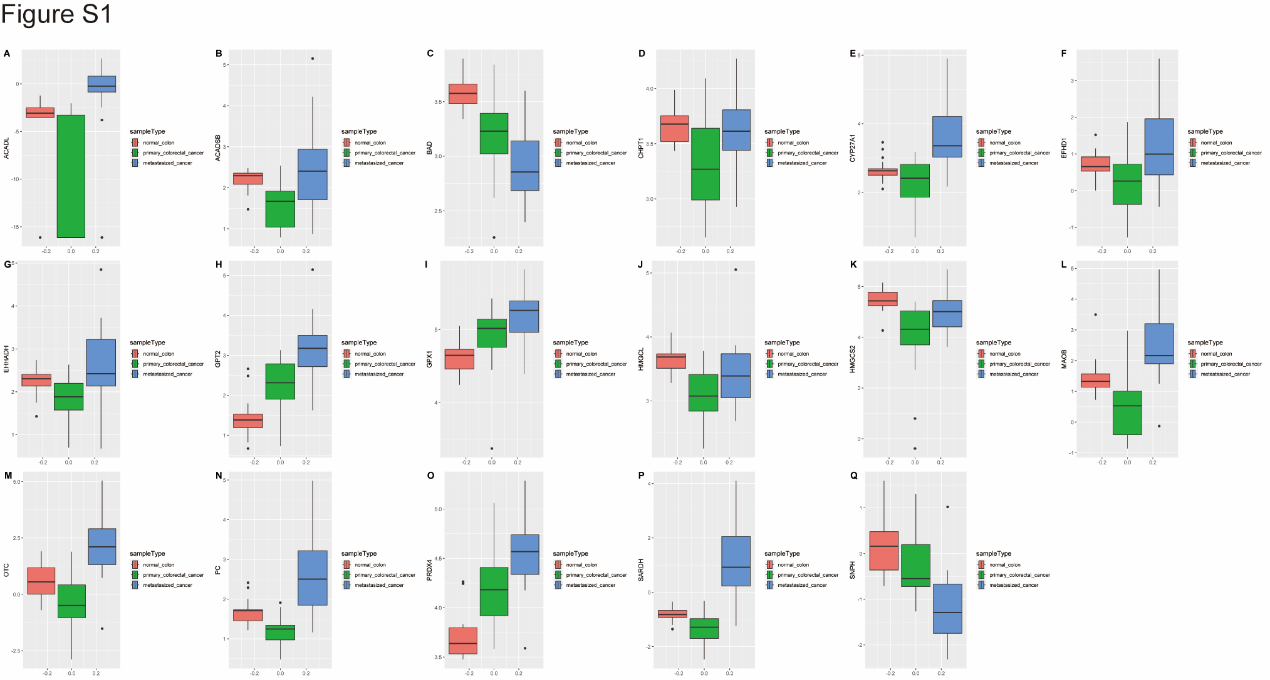
**

**Figure S1. A-Q. DEGs expression in paired normal, primary and metastasis samples in GSE50760.** *, *P*＜0.05; **, *P*＜0.01; *** *P*＜0.001; **** *P*＜0.0001.


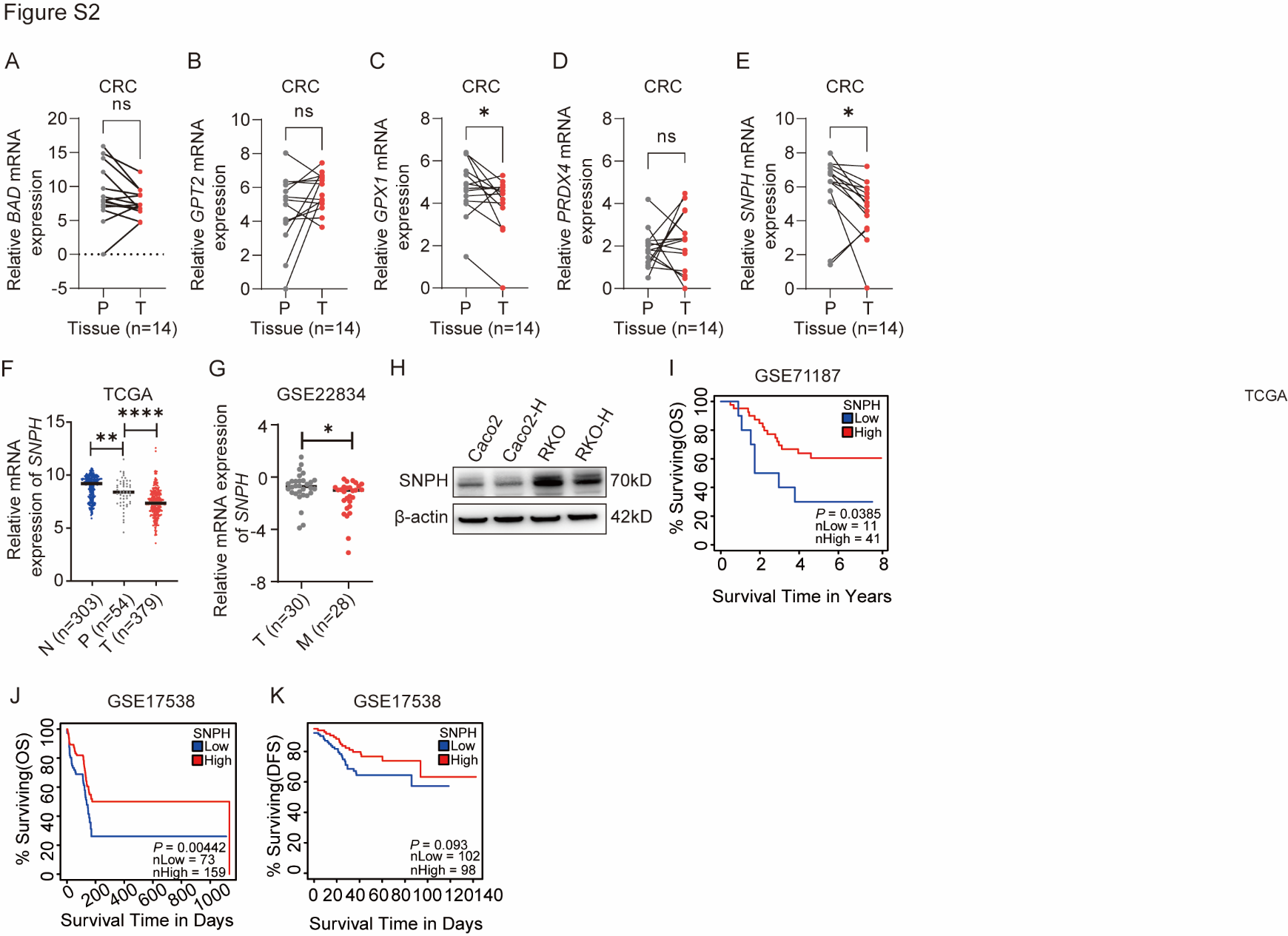
**Figure S2. SNPH is progressively down-regulated in colorectal cancer and liver metastases and significantly contributes to poor patient prognosis.** A-E. The relative expression of *BAD*, *GPT2*, *GPX1*, *PRDX4* and *SNPH* was measured by RT-qPCR in paired tissues from CRC patients. F-G. The relative expression of *SNPH* in paired tissues in TCGA-COAD and GSE22834. H. Western blot analyses for expression levels of SNPH in CRC cell lines Caco2, Caco2-H, RKO, RKO-H. I. Kaplan-Meier curve analysis of overall survival in CRC patients by the expression of SNPH in GSE71187. J-K. Kaplan-Meier curve analysis of overall survival (OS) and disease free survival (DFS) in CRC patients by the expression of SNPH in GSE17538. N, normal; P, peritumor; T, tumor; M, Liver metastasis. *, *P*＜0.05; **, *P*＜0.01; *** *P*＜0.001; **** *P*＜0.0001.

**
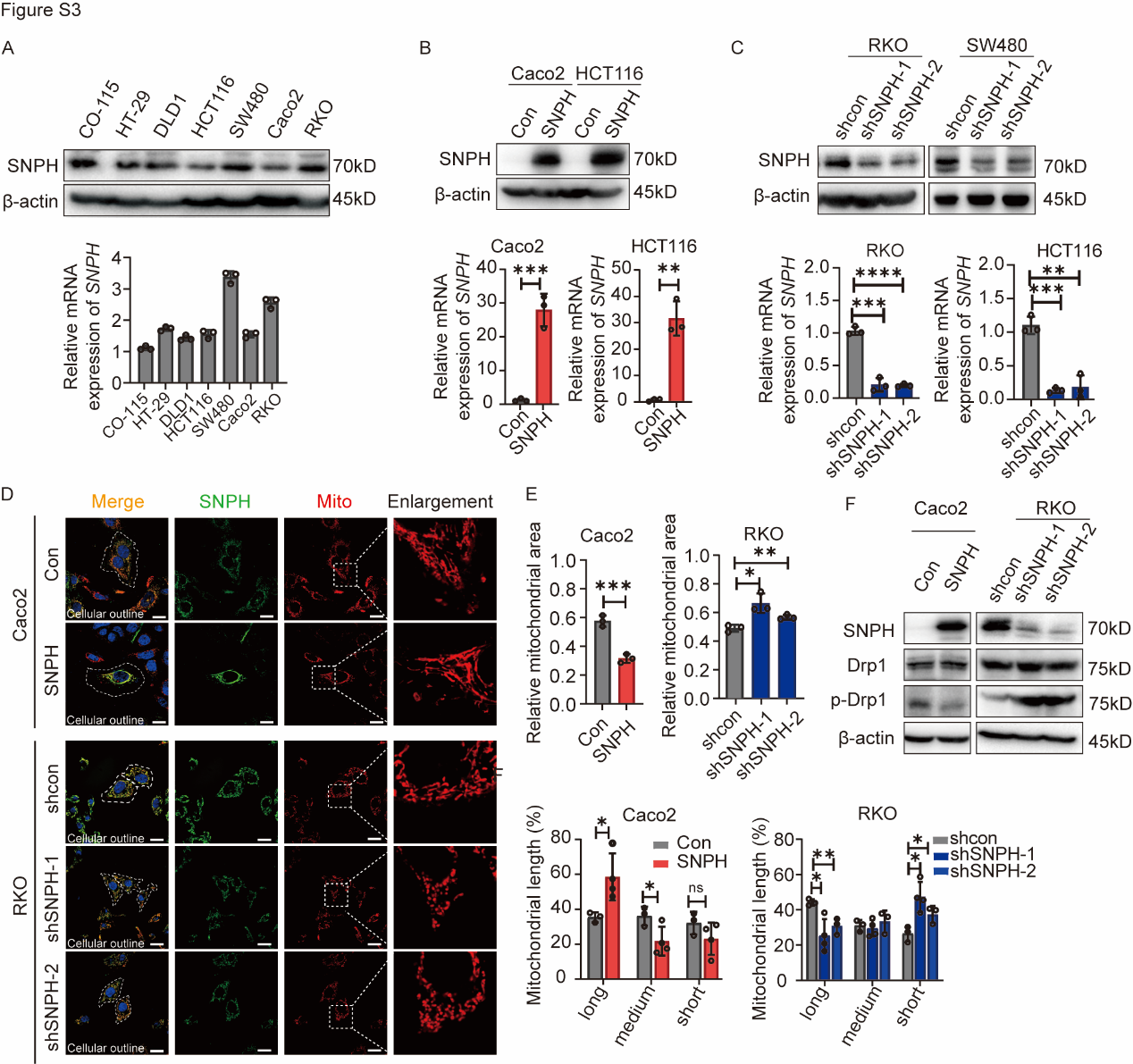
 Figure S3. Expression levels and effects on mitochondrial dynamics of SNPH in CRC cells.** A. qRT–PCR and western blot analyses SNPH expression in a panel of CRC cell lines. B-C. qRT–PCR and western blot analyses for SNPH expression were performed in Caco2 and HCT116 cells or RKO and SW480 cells, which were stably transfected with lentivirus vector as indicated. D-E. Representative images of IF staining for SNPH (green) and mitochondria (Mito-tracker-red) in CRC cells with different treatment as indicated. Scale bars, 5 µM. SNPH, expression vector encoding SNPH; Con, control vector; shSNPH-1 and shSNPH-2, shRNA against SNPH. F. Western blot analyses for Drp1 and p-Drp1 expression were performed in Caco2 and RKO cells, which were stably transfected with lentivirus vector as indicated.*, *P*＜0.05; **, *P*＜0.01; *** *P*＜0.001.

**
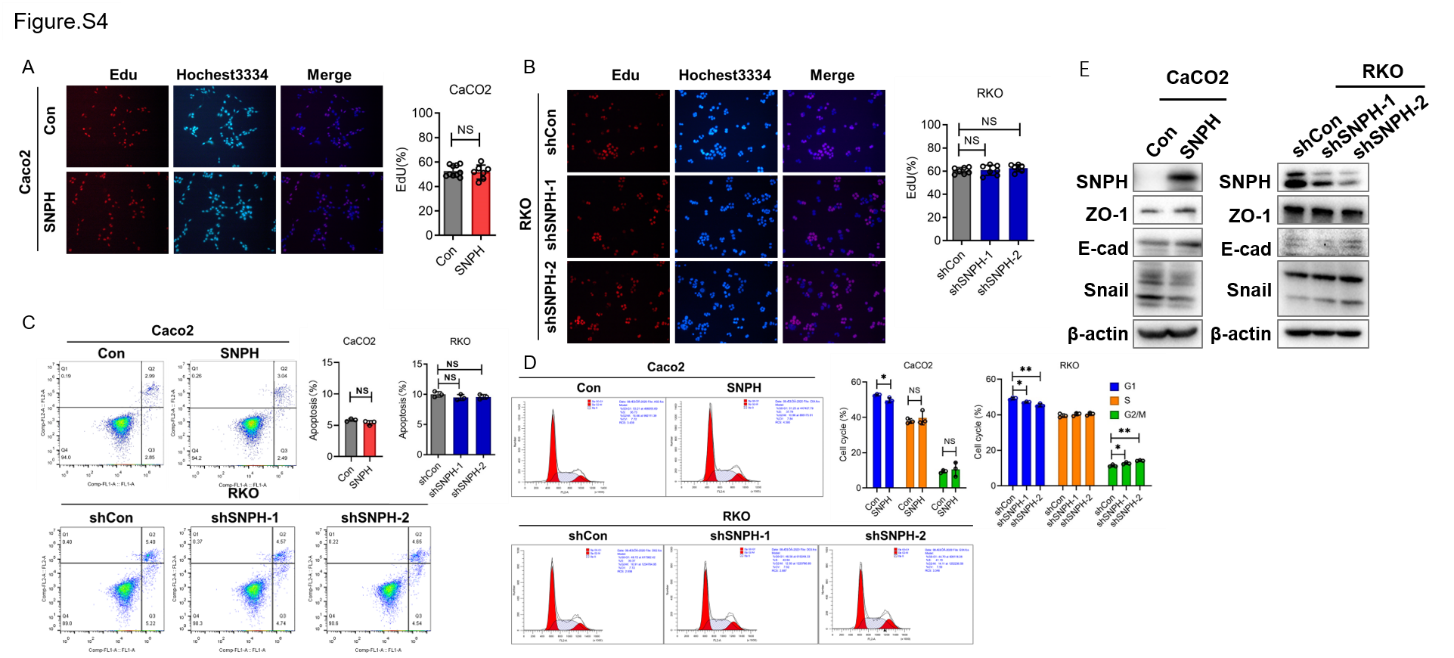
Figure S4. The effects of SNPH expression on cell proliferation, apoptosis, cell cycle and EMT marker in CRC.** A-B. Cell proliferation was evaluated by EdU incorporation assay in Caco2 and RKO cells as indicated. Scale bar, 50 μm. C. Flow cytometry analysis of apoptosis by ANXA5/Annexin V and PI staining in both Caco2 and RKO cells as indicated. D. Flow cytometry analysis of cell cycle by PI staining in both Caco2 and RKO cells as indicated. E. Western blot analyses for ZO-1, E-cad, Snail expression were performed in Caco2 and RKO cells as indicated.*, *P*＜0.05; **, *P*＜0.01.


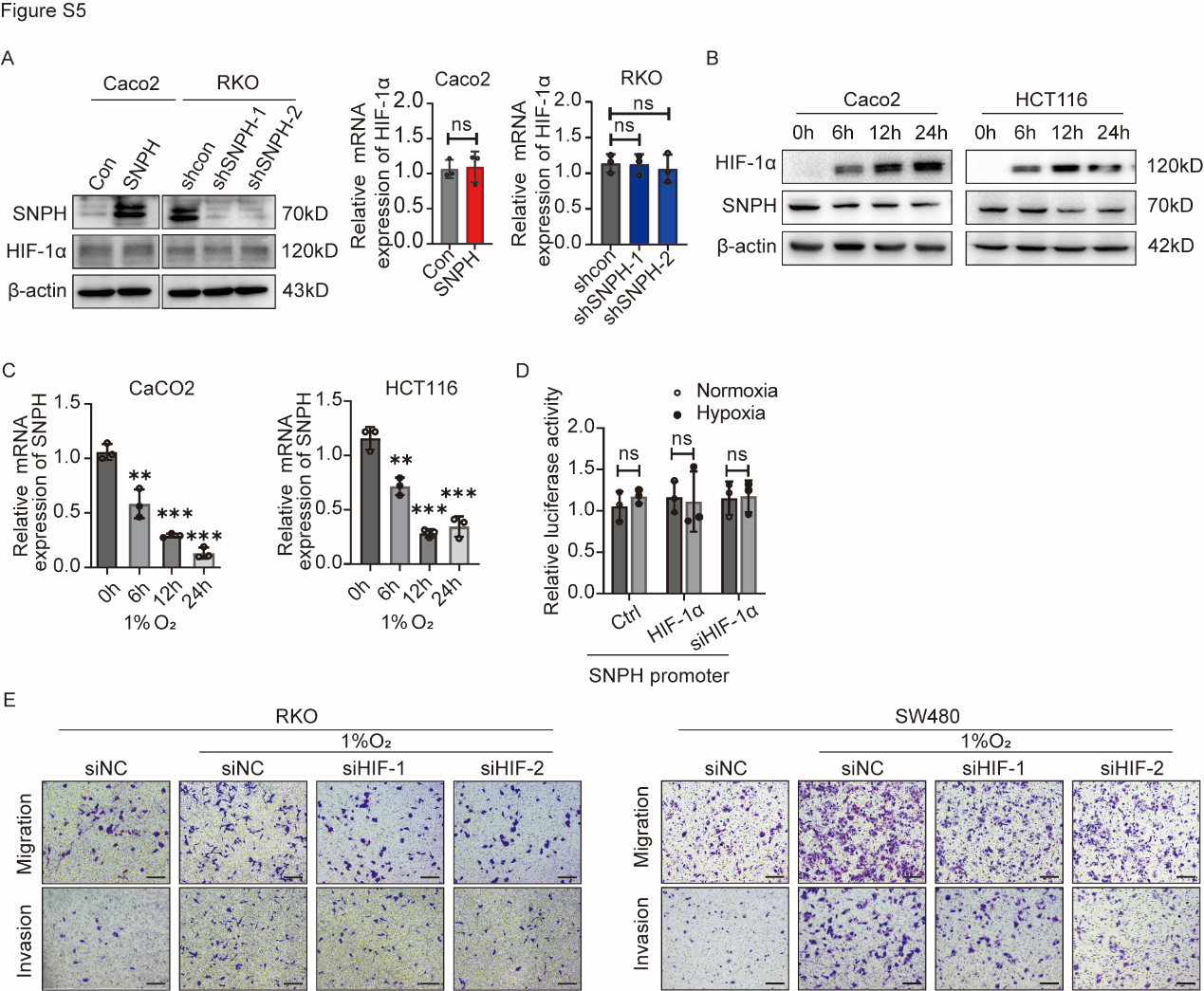


**Figure S5.** **HIF-1α-induced SNPH down-regulation promotes metastasis in CRC cells.**

A. qRT–PCR and western blot analyses for HIF-1α expression were performed in Caco2 and RKO cells, which were stably transfected with lentivirus vector as indicated. B-C. qRT–PCR and Western blot analyses for HIF-1α and SNPH expression were performed in Caco2 and HCT116 cells, which were treated with 1% O_2_ for different time. D. The luciferase activities of 293T after co-transfection with HIF-1α overexpression vector or siHIF-1α and luciferase reporter vectors p- mirGLO-SNPH-WT were examined. E. Transwell migration and invasion analysis for RKO and SW480 with treatment as indicated. *, *P*＜0.05; **, *P*＜0.01; *** *P*＜0.001.


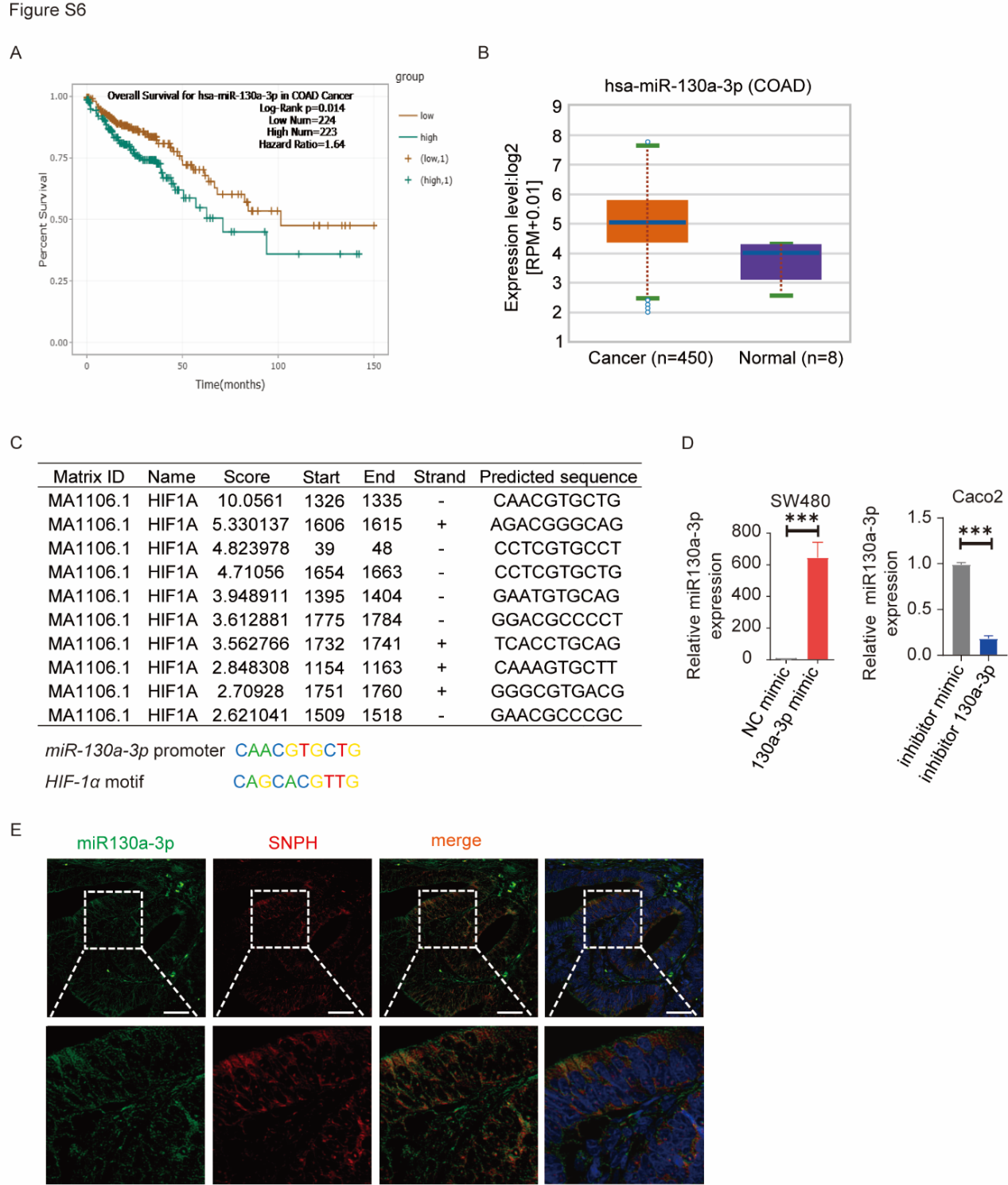


**Figure S6. miR-130a-3p expression and regulation in CRC tissues and their effects on prognosis of CRC patients.** A. Kaplan-Meier curve analysis of overall survival in CRC patients by the expression of miR-130a-3p in TCGA-COAD. B. The relative expression of miRNA-130a-3p in CRC tissues and adjacent normal tissues was analyzed in TCGA-COAD by GEPIA. C. The recognition interaction motif of HIF-1α and miR-130a-3p was predicted using the JSAPAR database. D. qRT–PCR analyses for miR-130a-3p expression were performed in SW480 and Caco2 cells as indicated. E. Representative images of RNAscope analysis of colorectal cancer tissues revealed co-localization of miRNA-130a-3p and SNPH. *, *P*＜0.05; **, *P*＜0.01; *** *P*＜0.001.

**
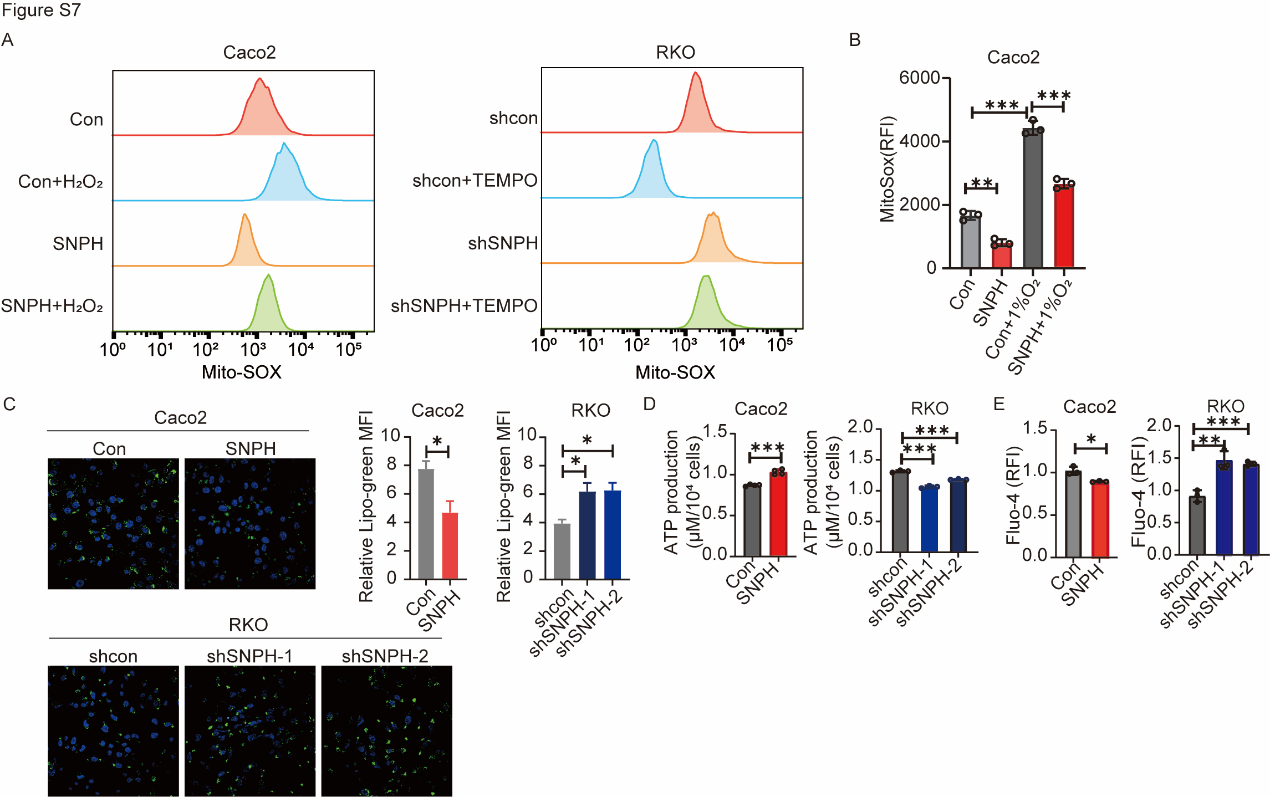
 Figure S7.** **SNPH regulation of ROS, lipid metabolism, calcium, and ATP generation in CRC cells.** A-B. Mitochondrial ROS levels were analyzed by flow cytometry in CRC cells treated as indicated. C. Lipid fluorescent probe staining in CRC cells treated as indicated, 200x. D. ATP generation analysis of CRC cells with treatmeant as indicated. E. Ca^2+^ levels were analyzed by flow cytometry in CRC cells treated as indicated. *, *P*＜0.05; **, *P*＜0.01; *** *P*＜0.001.**Supplementary Materials and methods**

- 1. **Ethynyl deoxyuridine (EdU) incorporation assay**

The proliferation of cells was analyzed using an EdU incorporation assay kit (Ribobio, China) according to the manufacturer’s instructions, and visualized under a fluorescent microscope.

- 1. **Cell cycle**

The cell cycle was established using PI staining (Keygentec, KGA511), according to the manufacturer's guidelines. The analysis of the cell cycle was carried out utilizing flow cytometry (Beckman, Fullerton, CA). The distinct phases of the cell cycle were identified based on the DNA content within the cells.

- 1. **Cell apoptosis analysis**

The detection of cell apoptosis was carried out using the ANXA5/Annexin V-FITC Apoptosis Detection Kit (BD, 556547), adhering to the manufacturer's recommended procedures. Finally, the prepared samples were analyzed using a flow cytometer (Beckman, Fullerton, CA).

- 1. **Luciferase Reporter Assay**

A total of 2.5×10^4^ CRC were co-transfected with HIF-1α overexpression vector or siHIF-1α and pmirGLO-SNPH-WT (Genepharma, China) and cultured in 5% CO_2_ for 24 hours. To assess the luciferase activity of the cell lysates, a Dual-Luciferase Reporter System (Promega, United States) was employed.

- 1. **RNAscope In Situ Hybridization**

Tissues were fixed, dehydrated, and subjected to antigen retrieval. After protease digestion, sections were hybridized with two target-specific probe (e.g., p-miR-130-3p-CY3 and p-ho-SNPH-FAM) at 40°C overnight. Cell nuclei were counterstained with DAPI. Fluorescent images were acquired using a Fluorescence microscope.

- 1. **Lipid staining**

BODIPY (MedChemExpress, HY-W090090) was utilized to monitor lipid in CRC cells. The cells were observed under the confocal microscope (Olympus FV 1000, Japan).

- 1. **ATP generation Assay**

Cells were seeded into a 96-well plate. ATP detection reagent (100 μL/well) was added to each well containing cells or ATP standards. Shaker for 2 minutes to ensure complete cell lysis，and incubated at room temperature for 10 minute. Luminescence was measured using a microplate reader. The ATP concentration was calculated based on the standard curve.

- 1. **Measurement of Cytosolic Ca2+ Concentration** The fluorescent dye Fluo-4/AM (Invitrogen) was used to monitor cytosolic Ca2+ concentration in living cells according to the manufacturer’s instructions. Briefly, cells were seeded in 6-well plate and loaded with 4 μM Fluo-4/AM, then washed with HBSS and followed by additional 30min incubation at 37℃ to allow complete de-esterification of intracellular AM (acetoxymethyl) esters before flow cytometer (Beckman, Fullerton, CA).

| **Table S1. The Relationship Between SNPH Expression and Clinicopathologic Features（N=117）** | | | | | |
| --- | --- | --- | --- | --- | --- |
| **Variables** | **Number of patients** | **SNPH expression** | | | **p-value** |
|  |  | **Low** | | **High** |  |
| **Total** | **117** | 73 | 44 | |  |
| **Age(years)** | |  |  | |  |
| ＞59 |  | 34 | 23 | | 0.5727 |
| ≤59 |  | 39 | 21 | |  |
| **Gender** |  |  |  | |  |
| Male |  | 49 | 27 | | 0.5535 |
| Female |  | 24 | 17 | |  |
| **Tumor Size** | |  |  | |  |
| ＞3.5cm |  | 35 | 25 | | 0.4454 |
| ≤3.5cm |  | 38 | 19 | |  |
| **CA199（U/ml）** |  |  |  | |  |
| ＞25 |  | 38 | 20 | | 0.5682 |
| ≤25 |  | 35 | 24 | |  |
| **CEA（U/ml）** |  |  |  | |  |
| ＞15 |  | 35 | 23 | | 0.7049 |
| ≤15 |  | 38 | 21 | |  |
| **Primary lesion site** | |  |  | |  |
| colon |  | 35 | 19 | | 0.7029 |
| Rectum |  | 38 | 25 | |  |
| **Lymphatic metastasis** | |  |  | |  |
| No |  | 30 | 15 | | 0.5568 |
| Yes |  | 43 | 29 | |  |
| **Pathological differentiation** | |  |  | |  |
| Well/Moderately | | 59 | 35 | | ＞0.9999 |
| Poorly/mucinous adenocarcioma | | 14 | 9 | |  |


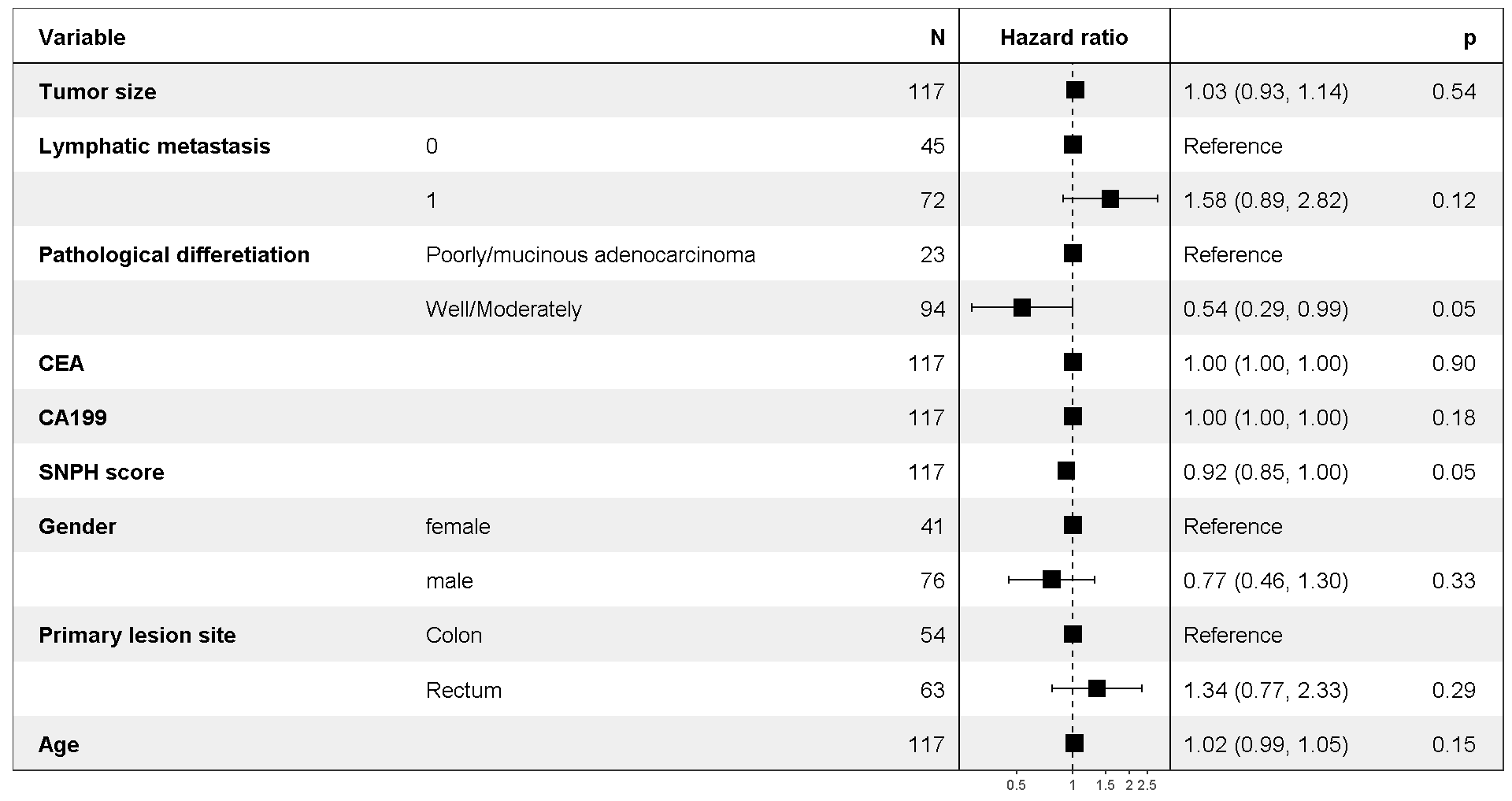
 **Table S2. Multivariable Cox regression of SNPH Expression and Clinicopathologic Features（N=117）**

| **Table S3. Sequence of primers, siRNA and RNAscope probe.** | | | |
| --- | --- | --- | --- |
| **1. Primers used in q-PCR analysis** | | | |
| BAD | Forward primer | CCCAGAGTTTGAGCCGAGTG | |
|  | Reverse primer | CCCATCCCTTCGTCGTCCT | |
| GPT2 | Forward primer | CCCATCCCACAATATCCCCTC | |
|  | Reverse primer | GTTCCCAGGGTTGATTATGCAG | |
| GPX1 | Forward primer | CAGTCGGTGTATGCCTTCTCG | |
|  | Reverse primer | GAGGGACGCCACATTCTCG | |
| PTDX4 | Forward primer | GCAAAGCGAAGATTTCCAAGC | |
|  | Reverse primer | CGCCAAAAGCGATAATTTCAGTT | |
| SNPH | Forward primer | CAGCAGCCGATGACACACT | |
|  | Reverse primer | CTGCACGAAGTCTGTCTGGA | |
| GAPDH | Forward primer | CTCTGCTCCTCCTGTTCGAC | |
|  | Reverse primer | GCGCCCAATACGACCAAATC | |
| miR-19a-3p | Forward primer | cgc ctg tgc aaa tct atg caa aac tga | |
| miR-19b-3p | Forward primer | ccg tgt gca aat cca tgc aaa act ga | |
| miR-130a-3p | Forward primer | ccg cag tgc aat gtt aaa agg gca t | |
| miR-130b-3p | Forward primer | cgc agt gca atg atg aaa ggg cat | |
| miR-301a-3p | Forward primer | gcg cag tgc aat agt att gtc aaa gc | |
| miR-301b-3p | Forward primer | gog cag tgc aat gat att gtc aaa gc | |
| miR-432a-3p | Forward primer | cgc cgt tgc ata gtc aca aaa gtg atc | |
| miR-454a-3p | Forward primer | cgc cgt agt gca ata ttg ctt ata ggg | |
| **2.siRNA** | | | |
| HIF-1α-1 | sense | UCAUCGACACUGUCAAGAATT | |
|  | antisense | UUCUUGACAGUGUCGAUGATT | |
| HIF-1α-2 | sense | GGAAAUGAGAGAAAUGCUUTT | |
|  | antisense | AAGCAUUUCUCUCAUUUCCTT | |
| siNC | sense | UUCUCCGAACGUGUCACGUTT | |
|  | antisense | ACGUGACACGUUCGGAGAATT | |
| **3.mimic RNA** | | | |
| mimic NC | sense | UUCUCCGAACGUGUCACGUTT | |
|  | antisense | ACGUGACACGUUCGGAGAATT | |
| miR-130a-3p mimic | sense | CAGUGCAAUGUUAAAAGGGCAU | |
|  | antisense | GCCCUUUUAACAUUGCACUGUU | |
| inhibitor mimic | 5`-3` | CAGUACUUUUGUGUAGUACAA | |
| inhibitor miR-130a-3p | 5`-3` | AUGCCCUUUUAACAUUGCACUG | |
| **4.RNAscope probe** | | |  |
| p-miR-130-3p | sense | aTGCCCTTTTaaCaTTGCaCTG | |
| p-ho-SNPH | 5`-3` | TCAATCTCTGTGTCCCGGTCCTGGAGCC | |

| **Table S4. Primary antibodies used for western blot, Immunofluorescence and immunohistochemistry.** | | |
| --- | --- | --- |
| **Antibody** | **Company (Cat. No.)** | **Working dilutions** |
| SNPH | abcam (ab192605) | WB 1:2000 IHC:1:1000 IF:100 |
| β-actin | CST(3700S) | WB 1:1000 |
| RAC1 | Sigma-Aldrich(NO.05389) | WB 1:1000 |
| cdc42 | Sigma-Aldrich(NO.05542) | WB 1:1000 |
| cdc42^GTP^ | NewEast Bio(26905) | IF:1:50 |
| PAK1 | abcam (223849) | WB 1:1000 |
| p-PAK1(Thr423) | CST (2601) | WB 1:1000 |
| Cofilin | CST (5175) | WB 1:1000 |
| p-Cofilin(ser3) | CST (3313) | WB 1:1000 |
| HIF-1α | BD (610959) | WB 1:1000 |
| HIF-1α | Genetech (MAB-0635) | IHC:Ready-to-Use |
| ERK | CST (4695S) | WB 1:1000 |
| p-ERK | CST (4370S) | WB 1:1000 |
| AKT | CST (9272S) | WB 1:1000 |
| p-AKT | CST (9271L) | WB 1:1000 |
| Ki67 | Servicebio (GB111499) | IHC:1:500 |
