# Supplementary material for: HIF-1α suppresses SNPH expression to facilitate liver metastasis of colorectal cancer through regulating mitochondrial dynamics and filopodia formation
Source: Cell Death Dis. 2026 Mar 26;17(1):380. doi: 10.1038/s41419-026-08551-1 (PMC13039215; doi:10.1038/s41419-026-08551-1)

Figure 1 G

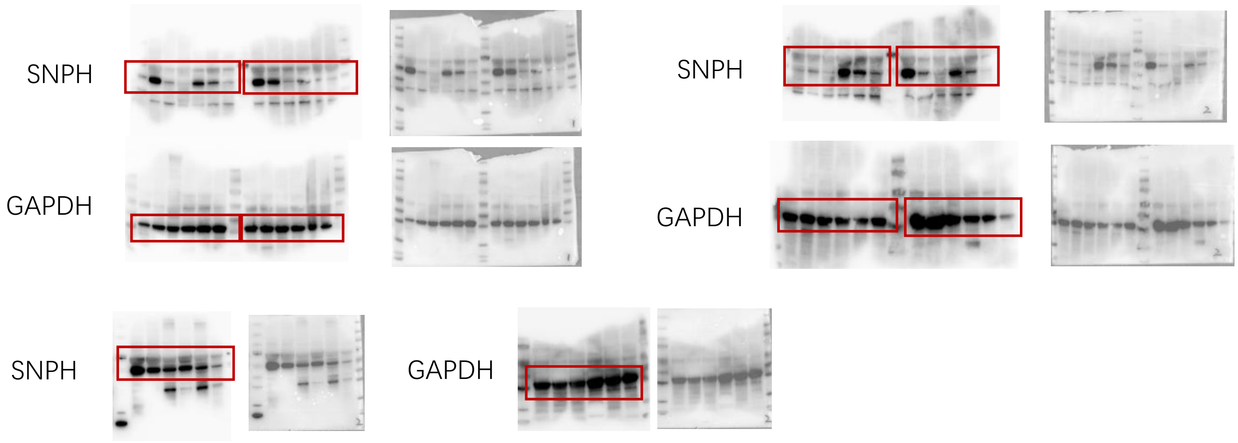

Figure 2 G

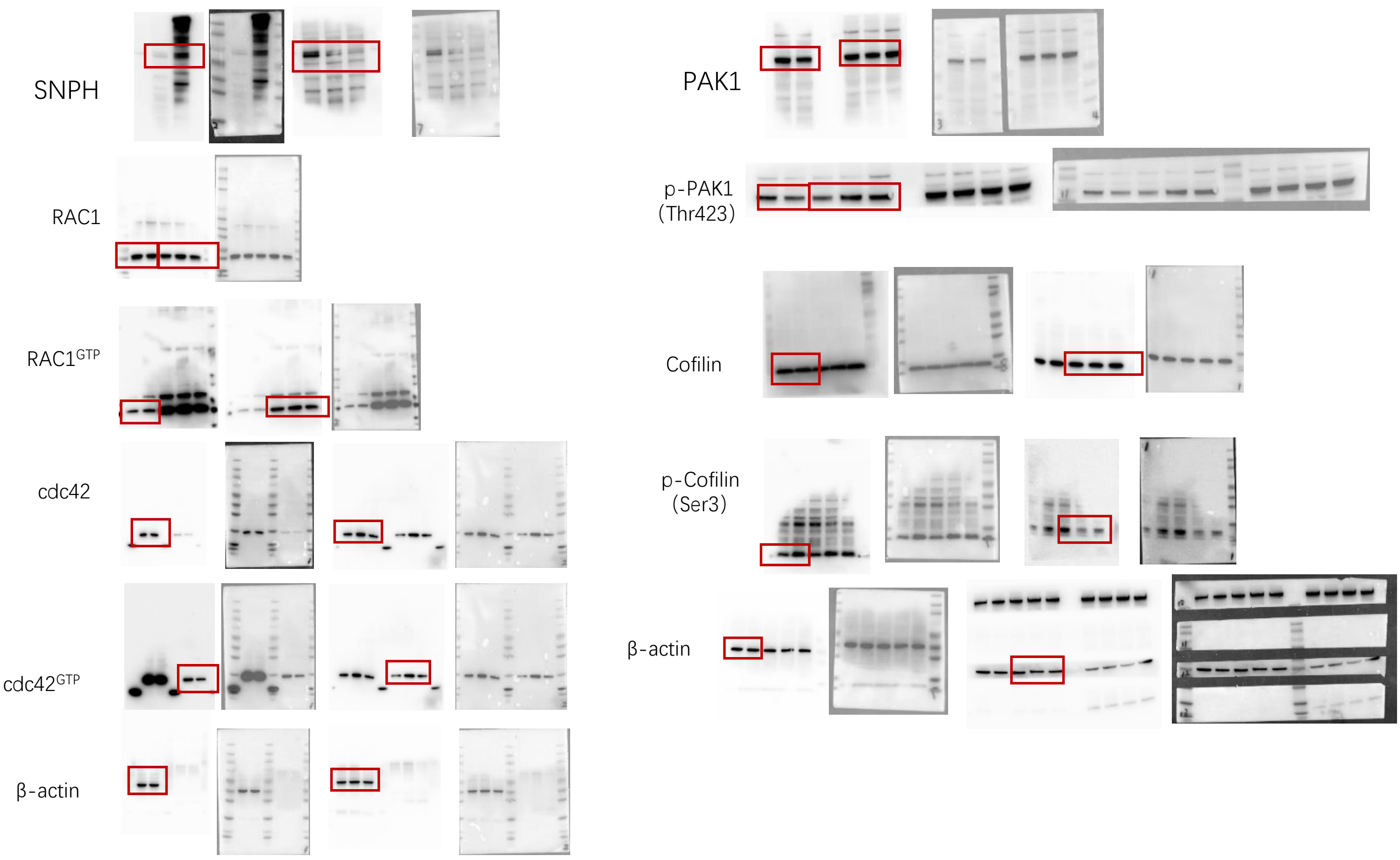

Figure 3 G

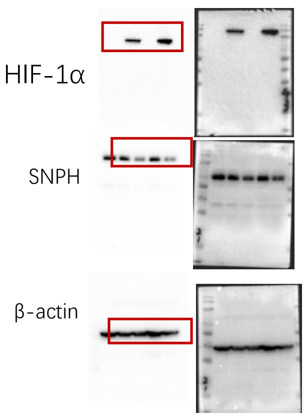

H

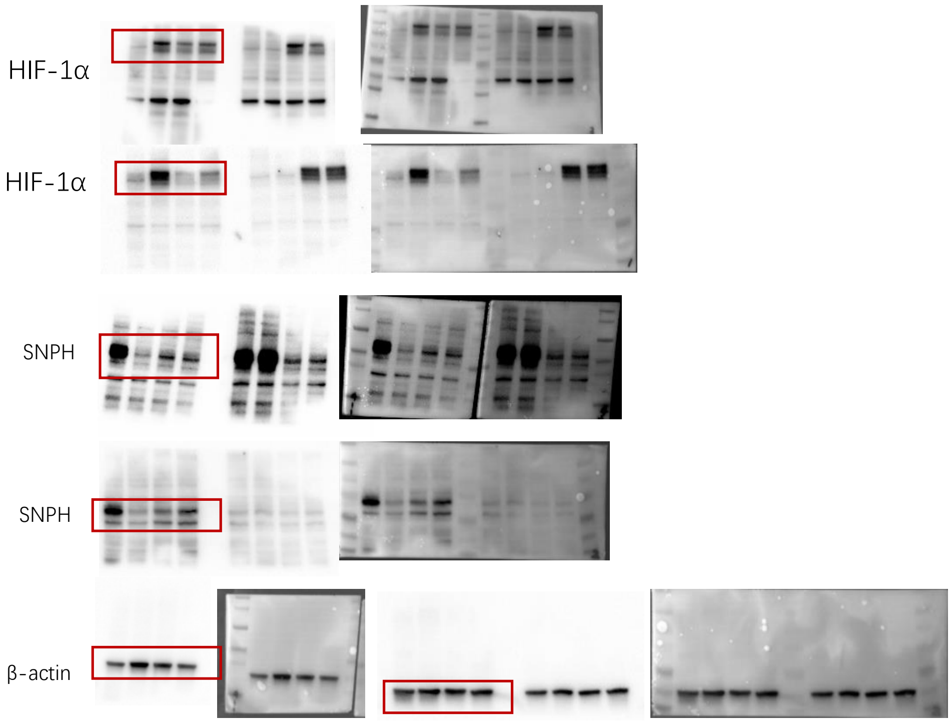

Figure 5 E

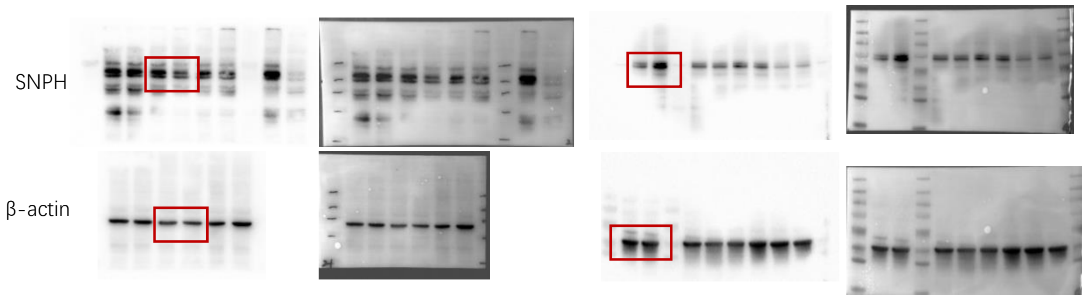

Figure 5 F

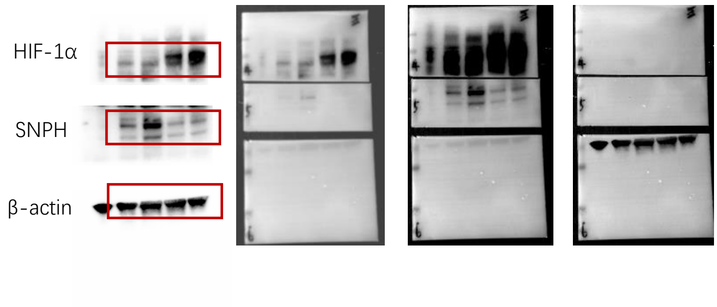

Figure 6 B

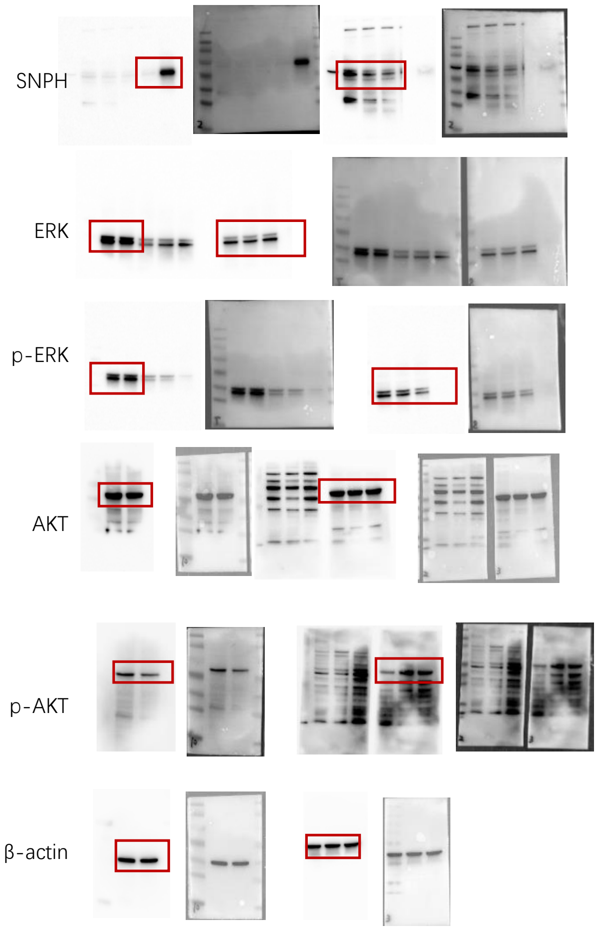

Figure 6 C

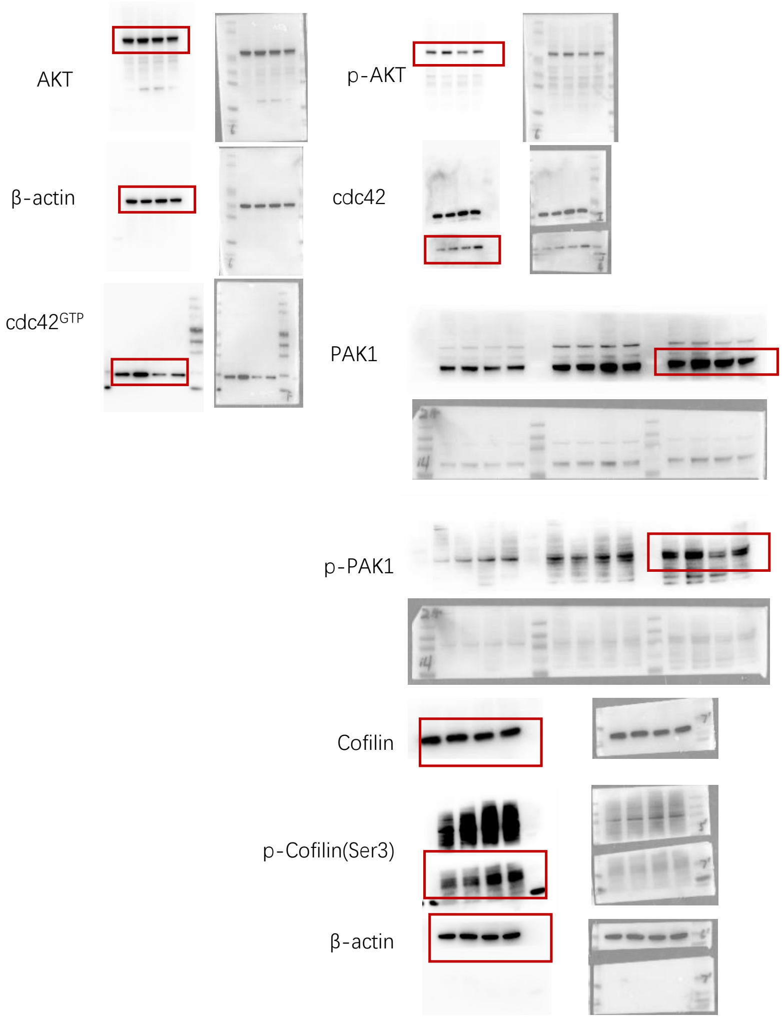

Figure 6 D

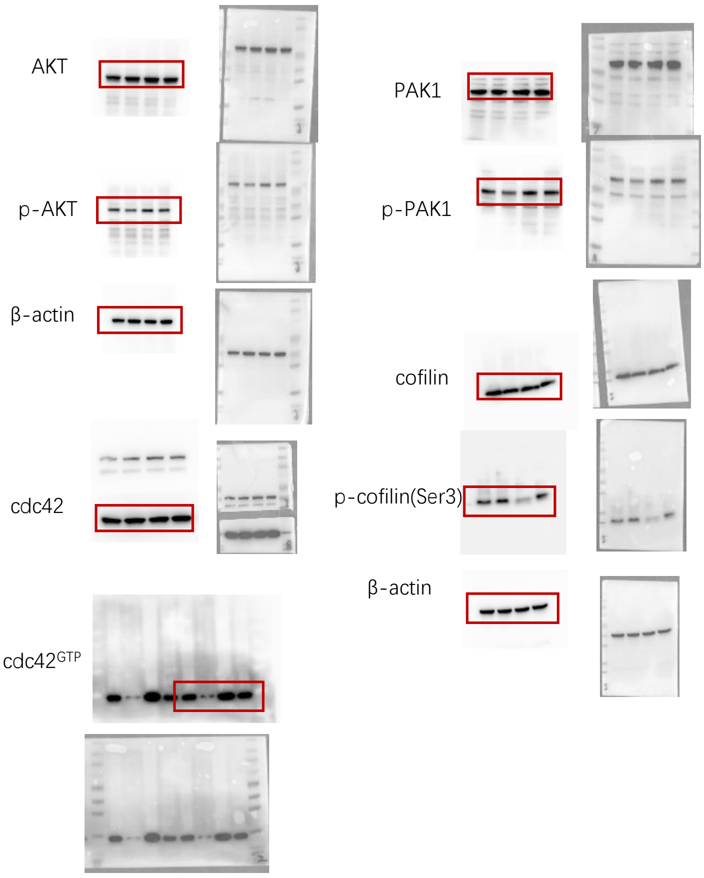

Figure 7 H

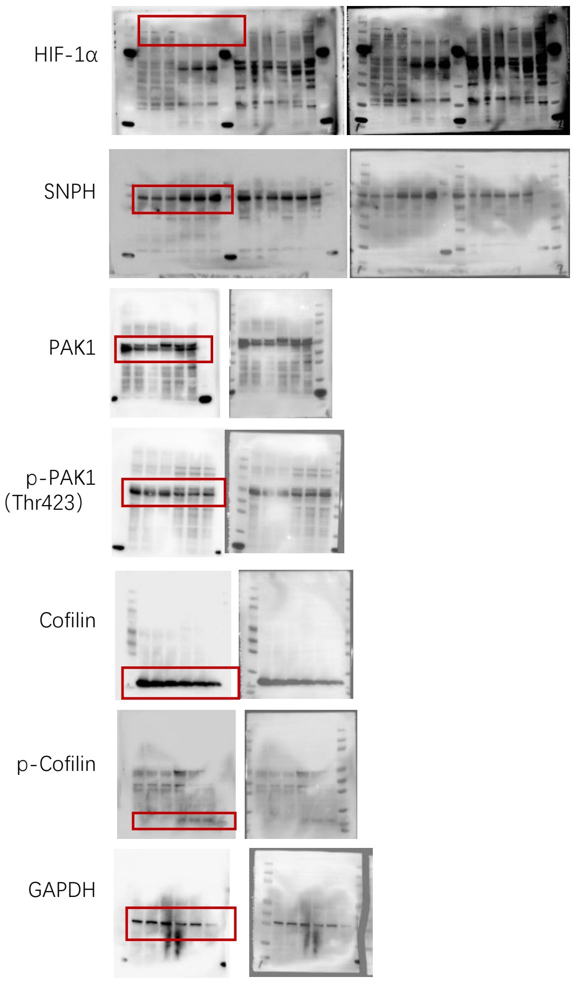

Figure S2 H

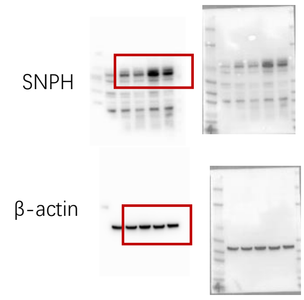

Figure S3

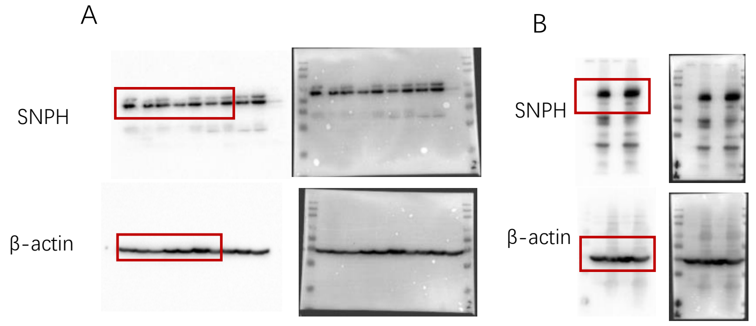

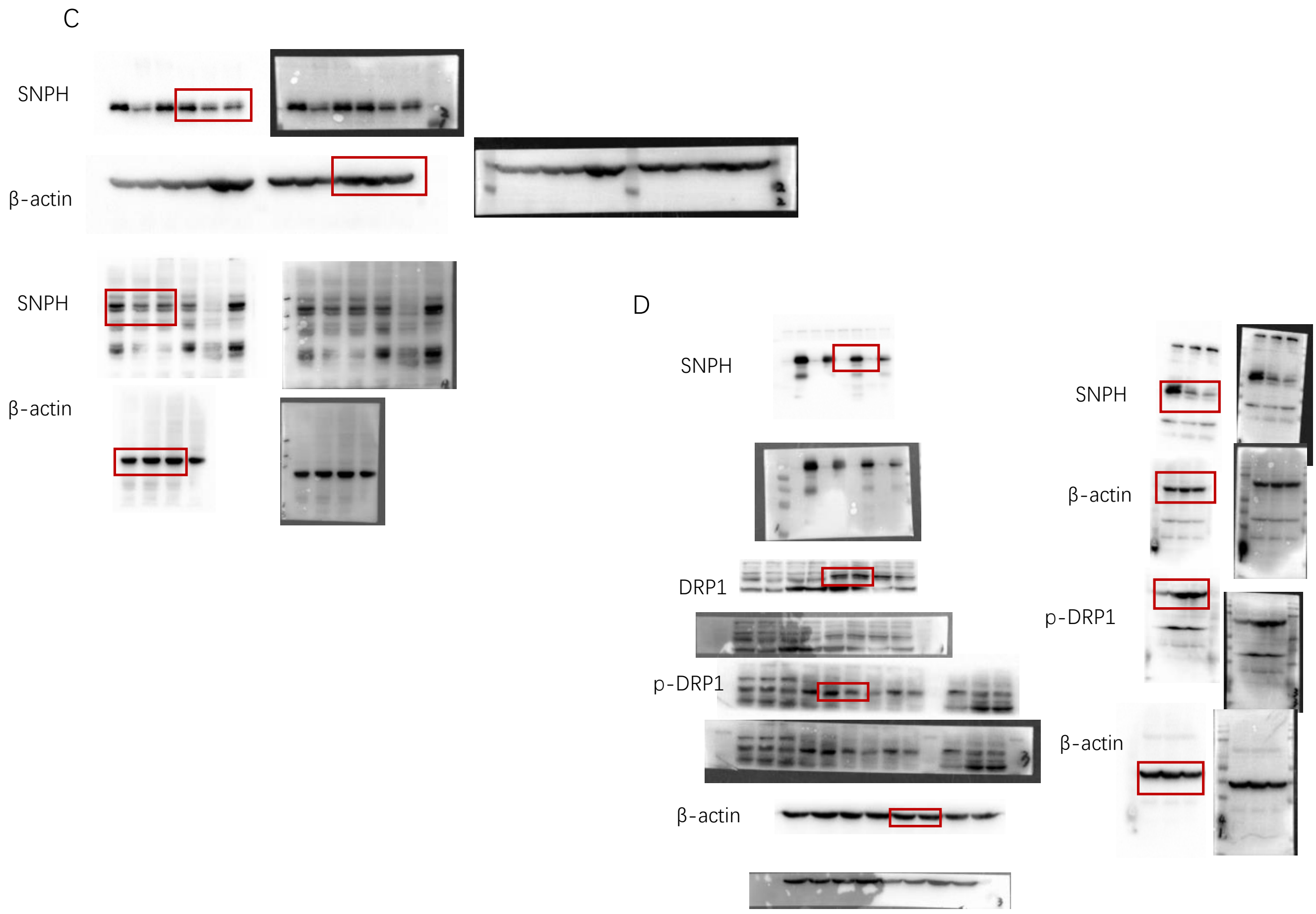

Figure S4

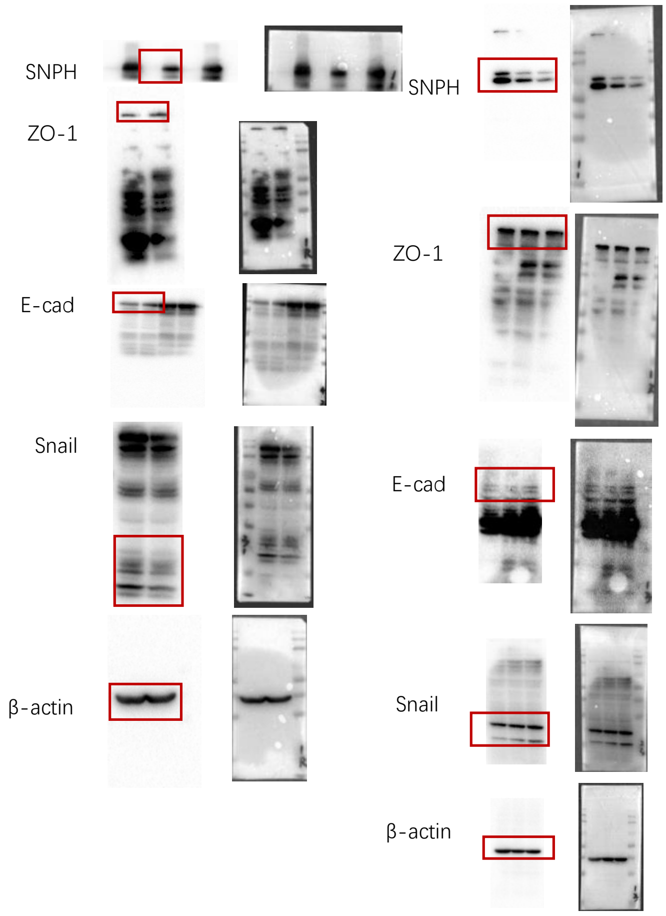

Figure S5

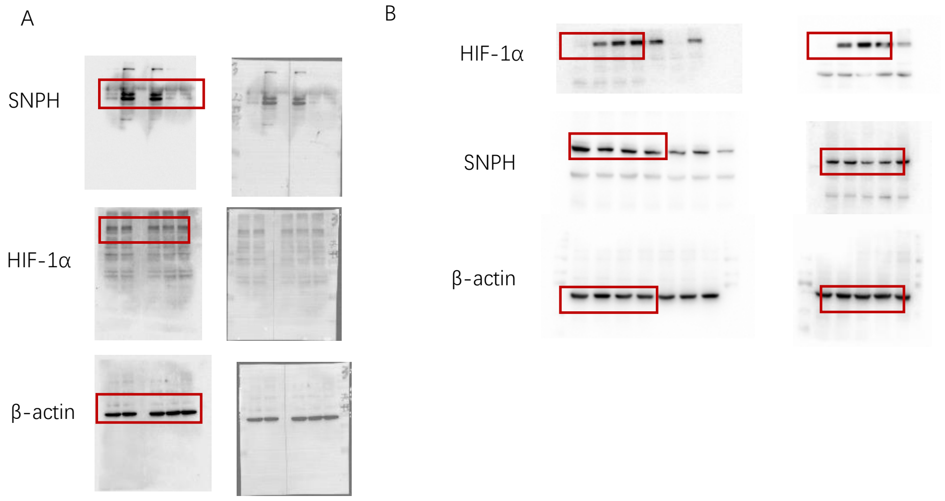

Supplement: Supplementary file 2 — Supplementary Material [file 41419_2026_8551_MOESM2_ESM.pdf]
